# Luminescent Pt(II) Complexes Using Unsymmetrical Bis(2-pyridylimino)isoindolate Analogues

**DOI:** 10.1021/acs.inorgchem.4c00558

**Published:** 2024-04-24

**Authors:** Ellie N. Payce, Richard C. Knighton, James A. Platts, Peter N. Horton, Simon J. Coles, Simon J. A. Pope

**Affiliations:** † School of Chemistry, Main Building, Cardiff University, Cardiff CF10 3AT, Cymru/Wales, U.K.; ‡ School of Chemistry, University of Southampton, Highfield, Southampton SO17 1BJ, England, U.K.; § UK National Crystallographic Service, Chemistry, Faculty of Natural and Environmental Sciences, University of Southampton, Highfield, Southampton SO17 1BJ, England, U.K.

## Abstract

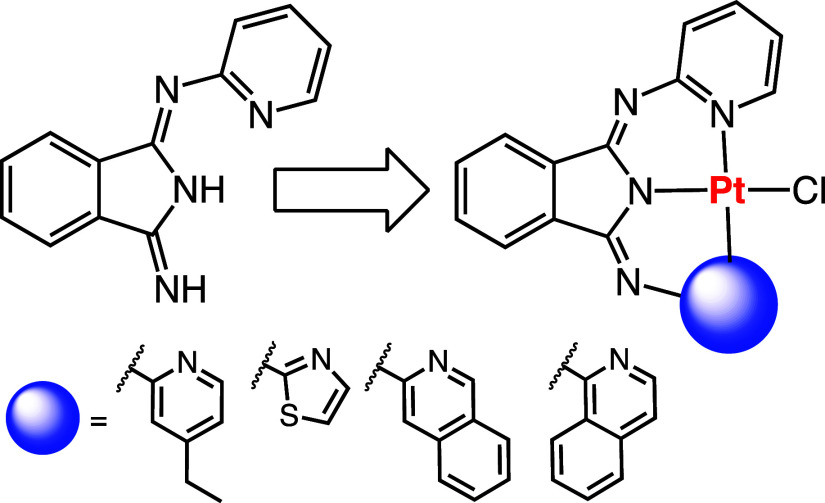

A series of ligands based upon a 1,3-diimino-isoindoline framework have been synthesized and investigated as pincer-type (N^∧^N^∧^N) chelates for Pt(II). The synthetic route allows different combinations of heterocyclic moieties (including pyridyl, thiazole, and isoquinoline) to yield new unsymmetrical ligands. Pt(**L**^**1–6**^)Cl complexes were obtained and characterized using a range of spectroscopic and analytical techniques: ^1^H and ^13^C NMR, IR, UV–vis and luminescence spectroscopies, elemental analyses, high-resolution mass spectrometry, electrochemistry, and one example via X-ray crystallography which showed a distorted square planar environment at Pt(II). Cyclic voltammetry on the complexes showed one irreversible oxidation between +0.75 and +1 V (attributed to Pt^2+/3+^ couple) and a number of ligand-based reductions; in four complexes, two fully reversible reductions were noted between −1.4 and −1.9 V. Photophysical studies showed that Pt(**L**^**1–6**^)Cl absorbs efficiently in the visible region through a combination of ligand-based bands and metal-to-ligand charge-transfer features at 400–550 nm, with assignments supported by DFT calculations. Excitation at 500 nm led to luminescence (studied in both solutions and solid state) in all cases with different combinations of the heterocyclic donors providing tuning of the emission wavelength around 550–678 nm.

## Abstract 2

A series of platinum(II) complexes have been synthesized using a range of unsymmetrical ligands based upon the bis(2-pyridylimino)isoindolate (BPI) framework. Structural, electrochemical, and electronic features have been investigated showing that deep red photoluminescent complexes can be obtained from a combination of different heterocyclic donors.

## Introduction

**Figure 1:**
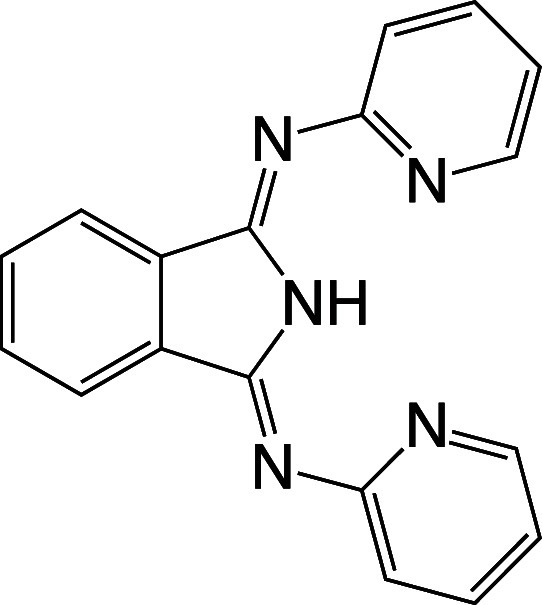
Molecular structure of bis(2-pyridylimino)isoindoline (BPI).

Bis(2-pyridylimino)isoindoline (BPI) (Figure 1) and its analogues continue to be an extremely important class of ligands in coordination chemistry. A potentially tridentate chelate, which typically binds as the anionic indolate form, has been alternatively described as a “pincer” class of ligand: a multitude of studies on its coordination chemistry have been reported with a range of metal ions.^1^ Complexes bearing the BPI-type ligand framework have been studied for >50 years, and applications include uses as dyes, chiral pincer ligands for catalysis, model systems of enzymes, and, as discussed below, in the development of metal-based luminophores.^2^

The pincer-like qualities of the BPI ligand framework have been exploited in catalytic systems based upon Pt(II) and Pd(II) for the photolytic activation of metal–carbon bonds.^3^ Ruthenium BPI complexes have also demonstrated catalytic activity in the dehydrogenation of alcohols and amines,^4^ and closely related Os(III) complexes were described previously by Gade and coworkers.^5^ Square planar Au(III) complexes of a BPI ligand functionalized with *tert*-butyl groups at the 4-positions of the pyridyl donors have also been described and have potential as precatalysts for organic transformations.^6^ While BPI and its variants are typically regarded as preferring, or at least imparting, a meridional binding mode, facial chelation modes within octahedral Re(I) complexes have also been recently reported.^7^

Recent interest in the BPI framework has been exemplified through the deployment of these ligand systems to promote metal-centered deep red to near-IR emission^8^ in octahedral Cr(III) complexes that can be tuned using different ligand variants.^9^ The rigidity of the ligand, combination of sigma- and pi-donating components, and imposition of a near-ideal octahedral geometry (through six-membered chelate rings) are specifically attractive features addressed by the BPI system that help optimize Cr(III)-centered photoluminescence.^10^

**Figure 2:**
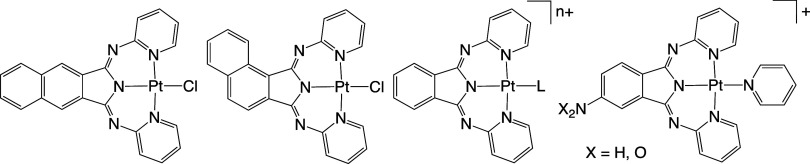
Relevant examples of Pt(II) complexes of BPI-related ligands demonstrate strategies for tuning emission. Left-to-right: site-specific variation in conjugation; changes in the auxiliary ligand (L); and use of substituents on isoindolate.

A number of Pt(II) complexes of BPI and its close variants (Figure 2) were reported about a decade ago and show that the resultant species are typically photoluminescent in the orange to red region of the visible spectrum. First, Chen and coworkers reported a series of complexes where the backbone of the BPI ligand was varied and the chloride auxiliary ligand was substituted for alkynyl ligands.^11^ The same group expanded the scope of the auxiliary ligands to other heterocycles, including substituted pyridines in an effort to tune emission wavelengths through an increasing contribution of ligand-centered triplet emission.^12^ The use of a bridging auxiliary ligand can also be employed to provide a route to linked dimetallic Pt(BPI)-based complexes.^13^ Thompson and Gordon and coworkers published closely related work at a similar time on the site-specific effects of conjugation on close variants of the BPI ligand framework and their resultant Pt(II) complexes;^14^ further studies also investigated the influence of different substituents positioned on the isoindolate unit.^15^

In general, the study of photoactive transition-metal complexes is an expanding area of chemistry and has important applications in many areas, such as light harvesting,^16−18^ sensing,^19,20^ energy upconversion,^21−23^ bioimaging,^24^ and theranostics.^25−27^ In all instances, an ability to tune the emission characteristics of the metal complexes, typically via modifications of the ligand architecture, is absolutely essential. It is in this context that we have revisited the use of BPI-type ligands for the development of luminescent Pt(II) complexes and present a new and simple synthetic methodology for obtaining unsymmetrical BPI analogues that allows different heterocyclic donors to be integrated into the ligand framework and thus has the potential for numerous iterations. The neutral Pt(L)Cl complexes emanating from these ligands demonstrate tunable luminescence features that are dictated by the structure and electronic nature of the ligand; the description of these complexes and their characterization are presented.

## Experimental Section

^1^H, ^13^C{^1^H} NMR spectra were recorded on an NMR-FT Bruker 500 MHz spectrometer in CDCl_3_. ^1^H and ^13^C{^1^H} NMR chemical shifts (δ) were determined relative to residual solvent peaks with digital locking and are given in parts per million. Coupling constants are given in hertz. High-resolution mass spectra were obtained by the staff at Cardiff University. UV–vis studies were performed on a Shimadzu UV-1800 spectrophotometer as MeCN solutions (1 × 10^–5^ M). Photophysical data were obtained on a JobinYvon–Horiba Fluorolog spectrometer fitted with a JY TBX picosecond photodetection module as CHCl_3_ solutions. The pulsed source was a Nano-LED configured for 295 nm output operating at 1 MHz or 500 kHz. Luminescence lifetime profiles were obtained using the JobinYvon–Horiba FluoroHub single-photon counting module, and the data fits yielded the lifetime values using the provided DAS6 deconvolution software. Quantum yield measurements were obtained using comparative actinometry on aerated MeCN solutions of the complexes using [Ru(bipy)_3_](PF_6_)_2_ in aerated MeCN as a standard (Φ = 0.018).^28^ The synthesis of **HL**^**5**^(8) and **HL**^**6**^(29) has been previously reported.

## Cyclic Voltammetry

Cyclic voltammetry was performed by using a PalmSens4 potentiostat. Experiments were performed using high-performance liquid chromatography-grade CH_2_Cl_2_ with an analyte concentration of 1 mM at 293 K using triply recrystallized [^*n*^Bu_4_N][PF_6_] as the supporting electrolyte at 0.1 M concentration. A three-electrode setup was used, consisting of a platinum disc working electrode, a platinum wire counter electrode, and a silver wire pseudoreference. Solutions were spared for 10 min with CH_2_Cl_2_-saturated stream of nitrogen gas. Voltammograms were referenced to the ferrocene/ferrocenium redox couple measured using the same conditions.

## Computational Methods

Electronic structure calculations were all performed using density functional theory within the Gaussian09 package.^30^ All calculations were performed using the PBE0^31^ functional and def2-SVP basis set and the corresponding effective core potential on platinum.^32^ Full geometry optimizations were performed on all complexes utilizing the self-consistent reaction field model which treats the solvent implicitly as a dielectric continuum. In all cases, the solvent chosen was chloroform, consistent with that utilized in synthesis and the majority of spectroscopic measurements. Minima were confirmed as stationary points through the computation of harmonic vibrational frequencies, which showed no imaginary components. These stationary points were used in single-point time-dependent DFT (TD-DFT) calculations to compute vertical excitation energies using the same basis set and functional. Decomposition of the molecular orbital character was performed using the GaussSum software package.^33^ Orbital plots used the Avogadro package.^34^

## X-ray Crystallography

Single orange rod-shaped crystals of **Pt(L**^**5**^**)Cl** were recrystallized from a mixture of hexane and chloroform, and data were collected following a standard method.^35^ A suitable crystal with dimensions 0.220 × 0.060 × 0.050 mm^3^ was selected and mounted on a MITIGEN holder in oil on a Rigaku FRE + diffractometer with ArcSec VHF Varimax confocal mirrors, a UG2 goniometer, and HyPix 6000HE detector. The crystal was kept at a steady *T* of 100(2) K during data collection. The structure was solved with the 2018/2 version of ShelXT solution program^36^ using dual methods and by using Olex2 1.5 as the graphical interface.^37^ The model was refined with 2018/3 version of ShelXL using full-matrix least-squares minimization on *F*^2^.^38^

## Synthesis

### Synthesis of *N*-(Pyridin-2-yl)isoindoline-1,3-diimine (**1**)

2-Aminopyridine (1.498 g, 15.9 mmol) was dissolved in dry tetrahydrofuran (THF) (60 mL) under an inert atmosphere. The solution was cooled in an ice bath, and NaH (0.417 g, 17.4 mmol) was added slowly. The light-brown solution was stirred at rt for 24 h, and phthalonitrile (1.856 g, 14.5 mmol) was subsequently added. After stirring at rt for a further 24 h, the solution was concentrated under reduced pressure and dissolved in MeOH (10 mL). Addition of H_2_O (80 mL) afforded the pure product as a precipitate, which was filtered and washed with H_2_O to give a pale-yellow solid (2.399 g, 75%). ^1^H NMR (500 MHz, CDCl_3_): δ_H_ 11.63 (1H, br s, NH), 8.45 (1H, ddd, *J*_HH_ = 4.9, 2.0, and 0.8 Hz, CH), 8.25 (1H, br s, NH), 8.01–8.10 (1H, m, CH), 7.70–7.92 (1H, m, CH), 7.75 (1H, ddd, *J*_HH_ = 8.1, 7.3, 2.0 Hz, CH), 7.60–7.70 (2H, m, CH), 7.43 (1H, dt, *J*_HH_ = 8.0, 1.0 Hz, CH), 7.09 (1H, ddd, *J*_HH_ = 7.3, 4.9, 1.1 Hz, CH). ^13^C{^1^H} NMR (126 MHz, CDCl_3_): δ_C_ 162.8, 160.9, 154.1, 147.4, 138.3, 136.9, 131.9, 131.7, 130.6, 123.3, 122.8, 121.3, 120.1. HRMS (ESI) [M + H]^+^: *m*/*z* 223.0984, calculated for [C_13_H_11_N_4_]^+^, measured 223.0990. IR (ATR, cm^–1^): ν_max_ 405, 473, 525, 544, 702, 748, 772, 779, 829, 887, 984, 1128, 1159, 1225, 1265, 1290, 1422, 1587, 1645, 2853 w, 2922 w, 2988 w, 3042 w, 3368 w. UV–vis (CHCl_3_) λ_max_ (ε, M^–1^ cm^–1^): 269 (12,674), 298 (7928), 311 (11,259), 338 (19,385), 353 (16,480), 373 (6669).

### Synthesis of *N*-(4-Ethylpyridin-2-yl)-*N*-(pyridin-2-yl)isoindoline-1,3-diimine (**HL**^**1**^)

**1** (0.295 g, 1.4 mmol) and 2-amino-4-ethylpyridine (0.165 g, 1.4 mmol) were dissolved in *n*-BuOH (15 mL) and were heated to reflux for 48 h. The solution was concentrated under reduced pressure and purified by flash column chromatography with dichloromethane (DCM)/MeCN (95:5 and 9:1) to afford a yellow oil (0.318 g, 72%). ^1^H NMR (500 MHz, CDCl_3_): δ_H_ 13.98 (1H, br s, NH), 8.60 (1H, ddd, *J*_HH_ = 4.8, 2.1, 0.8 Hz, CH), 8.49 (1H, d, *J*_HH_ = 5.1 Hz, CH), 8.07 (2H, dd, *J*_HH_ = 5.2, 3.3 Hz, CH), 7.74 (1H, td, *J*_HH_ = 7.7, 2.0 Hz, CH), 7.64 (2H, dd, *J*_HH_ = 5.6, 3.1 Hz, CH), 7.45 (1H, d, *J*_HH_ = 8.0 Hz, CH), 7.32 (1H, d, *J*_HH_ = 0.8 Hz, CH), 7.10 (1H, ddd, *J*_HH_ = 7.4, 4.8, 1.1 Hz, CH), 6.96 (1H, dd, *J*_HH_ = 5.0, 1.8 Hz, CH), 2.70 (2H, q, *J*_HH_ = 7.6 Hz, CH_2_), 1.29 (3H, t, *J*_HH_ = 7.6 Hz, CH_3_). ^13^C{^1^H} NMR (126 MHz, CDCl_3_): δ_C_ 160.7, 160.7, 155.4, 153.9, 153.8, 147.9, 147.7, 138.1, 135.9, 135.9, 131.7, 131.7, 123.3, 122.7, 122.6, 122.6, 120.4, 120.2, 28.3, 14.4. HRMS (ESI) [M + H]^+^: *m*/*z* 328.1562, calculated for [C_20_H_18_N_5_]^+^, measured 328.1551. IR (ATR, cm^–1^): ν_max_ 453, 482, 542, 619, 689, 704, 731, 772, 789, 828, 883, 912, 993, 1036, 1098, 1188, 1223, 1271, 1302, 1427, 1456, 1554, 1582, 1630, 2870 w, 2932 w, 2963 w, 3048 w, 3275 w. UV–vis (CHCl_3_) λ_max_ (ε, M^–1^ cm^–1^): 278 (22,209), 285 (21,588), 296 (21,211), 317 (15,076), 331 (18,737), 347 (20,028), 368 (23,155), 387 (24,986), 410 (14,176).

### Synthesis of *N*-(Pyridin-2-yl)-*N*-(thiazol-2-yl)isoindoline-1,3-diimine (**HL**^**2**^)

**1** (0.501 g, 2.3 mmol) and 2-aminothiazole (0.225 g, 2.3 mmol) were dissolved in *n*-BuOH (20 mL) and were heated to reflux for 48 h. The solution was concentrated under reduced pressure and purified by flash column chromatography with DCM/MeCN (99:1 and 95:5) to afford a yellow solid (0.183 g, 27%). ^1^H NMR (500 MHz, CDCl_3_): δ_H_ 13.80 (1H, br s, NH), 8.63 (1H, ddd, *J*_HH_ = 4.9, 2.0, and 0.8 Hz, CH), 8.05–8.10 (1H, m, CH), 8.00–8.05 (1H, m, CH), 7.78 (1H, td, *J*_HH_ = 7.7, and 2.0 Hz, CH), 7.75 (1H, d, *J*_HH_ = 3.6 Hz, CH), 7.60–7.69 (2H, m, CH), 7.47 (1H, d, *J*_HH_ = 8.0 Hz, CH), 7.17 (1H, d, *J*_HH_ = 3.6 Hz, CH), 7.14 (1H, ddd, *J*_HH_ = 7.4, 4.9, 1.1 Hz, CH). ^13^C{^1^H} NMR (126 MHz, CDCl_3_): δ_C_ 172.4, 160.2, 153.7, 153.1, 148.1, 140.9, 138.3, 136.2, 134.9, 132.1, 131.9, 123.5, 122.9, 122.9, 120.7, 117.3. HRMS (ESI) [M + H]^+^: *m*/*z* 306.0813, calculated for [C_16_H_12_N_5_S]^+^, measured 306.0802. IR (ATR, cm^–1^): ν_max_ 419, 503, 532, 608, 704, 737, 748, 777, 793, 847, 876, 1040, 1099, 1125, 1142, 1190, 1215, 1248, 1300, 1375, 1431, 1456, 1555, 1578, 1620, 1643, 2980 w, 3067 w, 3198 w. UV–vis (CHCl_3_) λ_max_ (ε, M^–1^ cm^–1^): 289 (21,113), 300 (18,957), 329 (17,037), 343 (20,151), 361 (21,064), 385 (24,826), 406 (24,014), 432 (12,267).

### Synthesis of *N*-(Isoquinolin-3-yl)-*N*-(pyridin-2-yl)isoindoline-1,3-diimine (**HL**^**3**^)

**1** (0.500 g, 2.3 mmol) and 3-aminoisoquinoline (0.325 g, 2.3 mmol) were dissolved in *n*-BuOH (15 mL) and were heated to reflux for 48 h. The golden solution was concentrated under reduced pressure, and addition of MeOH (20 mL)/H_2_O (20 mL) gave a precipitate, which was filtered and washed with H_2_O to afford a yellow solid (0.524 g, 67%). ^1^H NMR (500 MHz, CDCl_3_): δ_H_ 14.01 (1H, br s, NH), 9.27 (1H, s, CH), 8.68 (1H, dd, *J*_HH_ = 4.9, 2.0 Hz, CH), 8.10–8.13 (1H, m, CH), 8.06–8.10 (1H, m, CH), 7.99 (1H, d, *J*_HH_ = 8.2 Hz, CH), 7.85 (1H, d, *J*_HH_ = 8.6 Hz, CH), 7.83 (1H, s, CH), 7.77 (1H, td, *J*_HH_ = 7.7, 2.0 Hz, CH), 7.60–7.70 (3H, m, CH), 7.54 (1H, t, *J*_HH_ = 7.5 Hz, CH), 7.47 (1H, d, *J*_HH_ = 7.9 Hz, CH), 7.13 (1H, ddd, *J*_HH_ = 7.4, 4.8, 1.1 Hz, CH). ^13^C{^1^H} NMR (126 MHz, CDCl_3_): δ_C_ 160.8, 155.3, 153.9, 152.6, 151.1, 147.9, 138.1, 138.1, 136.0, 135.8, 131.7, 131.5, 130.7, 127.8, 127.1, 126.8, 126.8, 123.3, 122.7, 122.6, 120.1, 118.5. HRMS (ESI) [M + H]^+^: *m*/*z* 350.1406, calculated for [C_22_H_16_N_5_]^+^, measured 350.1397. IR (ATR, cm^–1^): ν_max_ 465, 478, 673, 687, 704, 743, 772, 787, 880, 951, 1034, 1099, 1138, 1188, 1231, 1275, 1427, 1456, 1553, 1574, 1614, 1634, 2853 w, 2922 w, 3053 w, 3291 w. UV–vis (CHCl_3_) λ_max_ (ε, M^–1^ cm^–1^): 279 (20,825), 291 (18,561), 304 (16,775), 343 (20,486), 350 (19,968), 359 (19,269), 385 (21,729), 406 (24,063), and 430 (13,870).

### Synthesis of *N*-(Isoquinolin-1-yl)-*N*-(pyridin-2-yl)isoindoline-1,3-diimine (**HL**^**4**^)

**1** (0.500 g, 2.3 mmol) and 1-aminoisoquinoline (0.325 g, 2.3 mmol) were dissolved in *n*-BuOH (15 mL) and were heated to reflux for 48 h. The yellow-brown solution was concentrated under reduced pressure, and addition of MeOH (20 mL)/H_2_O (20 mL) gave a precipitate, which was filtered and washed with H_2_O to afford a golden-yellow solid (0.260 g, 33%). ^1^H NMR (500 MHz, CDCl_3_): δ_H_ 14.15 (1H, br s, NH), 9.01 (1H, d, *J*_HH_ = 8.3 Hz, CH), 8.66 (1H, ddd, *J*_HH_ = 4.8, 2.1, 0.8 Hz, CH), 8.50 (1H, d, *J*_HH_ = 5.7 Hz, CH), 8.20–8.25 (1H, m, CH), 8.08–8.13 (1H, m, CH), 7.74–7.84 (2H, m, CH), 7.62–7.73 (4H, m, CH), 7.49 (1H, s, CH), 7.47 (1H, d, *J*_HH_ = 2.4 Hz, CH), 7.14 (1H, ddd, *J*_HH_ = 7.4, 4.8, 1.1 Hz, CH). ^13^C{^1^H} NMR (126 MHz, CDCl_3_): δ_C_ 160.6, 158.7, 154.4, 154.0, 148.0, 141.0, 138.2, 137.8, 136.2, 136.0, 131.9, 131.8, 130.5, 127.2, 127.1, 126.6, 126.3, 123.4, 122.9, 122.8, 120.4, 118.3. HRMS (ESI) [M + H]^+^: *m*/*z* 350.1406, calculated for [C_22_H_16_N_5_]^+^, measured 350.1396. IR (ATR, cm^–1^): ν_max_ 482, 544, 557, 579, 644, 664, 675, 702, 737, 772, 779, 795, 1015, 1024, 1096, 1144, 1184, 1204, 1221, 1298, 1344, 1425, 1454, 1493, 1547, 1574, 1609, 2853 w, 2922 w, 2955 w, 3046 w, 3194 w, 3229 w. UV–vis (CHCl_3_) λ_max_ (ε, M^–1^ cm^–1^): 283 (17,994), 294 (18,336), 325 (14,081), 333 (13,999), 349 (14,674), 367 (14,876), 397 (17,694), 418 (15,134), 443 (6721).

### Synthesis of Pt(**L**^**1**^)Cl

**HL**^**1**^ (0.045 g, 0.14 mmol) and Pt(COD)Cl_2_ (0.056 g, 0.15 mmol) were dissolved in MeOH (12 mL) and heated to reflux for 24 h. The yellow solution rapidly changed color upon addition of N,N-diisopropylethylamine (DIPEA) (28.3 μL, 0.15 mmol) giving a red precipitate. The solid was filtered, washed with MeOH, and dissolved in toluene to afford a red solid (0.050 g, 66%). ^1^H NMR (500 MHz, CDCl_3_): δ_H_ 10.30 (1H, dd, *J*_HH_ = 6.5, 1.7 Hz, CH), 10.14 (1H, d, *J*_HH_ = 6.7 Hz, CH), 8.02–8.11 (2H, m, CH), 7.88–7.95 (1H, m, CH), 7.61–7.66 (2H, m, CH), 7.60 (1H, dd, *J*_HH_ = 8.1, 1.7 Hz, CH), 7.45 (1H, d, *J*_HH_ = 2.3 Hz, CH), 7.02 (1H, td, *J*_HH_ = 6.8, 1.8 Hz, CH), 6.88 (1H, dd, *J*_HH_ = 6.6, 2.4 Hz, CH), 2.70 (2H, q, *J*_HH_ = 7.6 Hz, CH_2_), 1.34 (3H, t, *J*_HH_ = 7.6 Hz, CH_3_). ^13^C{^1^H} NMR (126 MHz, CDCl_3_): δ_C_ 156.1, 152.6, 152.0, 151.3, 151.3, 150.2, 149.8, 138.2, 137.7, 137.7, 131.5, 131.5, 127.6, 126.3, 122.4, 122.4, 120.5, 119.8, 28.0, 13.7. Elemental analysis: found, C 42.93%, H 2.45%, N 12.05%; calculated for C_20_H_16_N_5_PtCl, C 43.13%, H 2.90%, N 12.58%. HRMS (ESI) [M + H]^+^: *m*/*z* 556.0799, calculated for [C_20_H_17_N_5_^194^PtCl]^+^, measured 556.0802. IR (ATR, cm^–1^): ν_max_ 419, 482, 696, 743, 766, 822, 878, 914, 1018, 1099, 1184, 1296, 1381, 1418, 1431, 1460, 1516, 1535, 1578, 1584, 1649, 2880 w, 2965 w, 3121 w. UV–vis (CHCl_3_) λ_max_ (ε, M^–1^ cm^–1^): 277 (33,553), 348 (19,903), 386 (10,956), 472 (12,422), 491 (12,333).

### Synthesis of Pt(L^2^)Cl

The same procedure as Pt(**L**^**1**^)Cl, except **HL**^**2**^ (0.050 g, 0.16 mmol), Pt(COD)Cl_2_ (0.068 g, 0.18 mmol), and DIPEA (27.9 μL, 0.18 mmol), was used. The solid was filtered, washed with MeOH, and dissolved in toluene to afford a bright red solid (0.041 g, 47%). ^1^H NMR (500 MHz, CDCl_3_): δ_H_ 10.48 (1H, dd, *J*_HH_ = 6.5, 1.7 Hz, CH), 9.32 (1H, d, *J*_HH_ = 4.4 Hz, CH), 8.13–8.17 (1H, m, CH), 8.07–8.12 (1H, m, CH), 8.00 (1H, ddd, *J*_HH_ = 8.4, 7.0, 1.7 Hz, CH), 7.73 (1H, dd, *J*_HH_ = 8.0, 1.8 Hz, CH), 7.63–7.71 (2H, m, CH), 7.13 (1H, td, *J*_HH_ = 6.7, 1.8 Hz, CH), 7.07 (1H, d, *J*_HH_ = 4.4 Hz, CH). Elemental analysis: found, C 36.28%, H 2.44%, N 12.37%; calculated for C_16_H_11_N_5_SPtCl.0.5MeOH, C 35.91%, H 2.37%, N 12.69%. HRMS (ESI) [M + H]^+^: *m*/*z* 536.0073, calculated for [C_16_H_11_N_5_S^196^PtCl]^+^, measured 536.0063. IR (ATR, cm^–1^): ν_max_ 419, 521, 637, 704, 750, 770, 924, 1107, 1117, 1192, 1207, 1391, 1468, 1510, 1533, 1562, 1643, 2980 w, 3063 w, 3127 w. UV–vis (CHCl_3_) λ_max_ (ε, M^–1^ cm^–1^): 341 (15,438), 354 (15,660), 383 (12,836), 471 (11,179), 497 (10,815).

### Synthesis of Pt(**L**^**3**^)Cl

**HL**^**3**^ (0.034 g, 0.10 mmol) and Pt(COD)Cl_2_ (0.040 g, 0.11 mmol) were dissolved in MeOH/DCM (15:5 mL) and heated to reflux for 24 h. The addition of DIPEA (18.4 μL, 0.11 mmol) afforded a red precipitate, which was filtered, washed with MeOH, and dried to afford a bright red solid (0.038 g, 68%). ^1^H NMR (500 MHz, CDCl_3_): δ_H_ 11.15 (1H, s, CH), 10.25 (1H, dd, *J*_HH_ = 6.4, 1.8 Hz, CH), 8.04–8.13 (3H, m, CH), 8.02 (1H, s, CH), 7.79–7.93 (3H, m, CH), 7.55–7.67 (4H, m, CH), 7.00 (1H, td, *J*_HH_ = 6.7, 1.8 Hz, CH). Elemental analysis: found, C 46.34%, H 2.31%, N 11.77%; calculated for C_22_H_14_N_5_PtCl, C 45.64%, H 2.44%, N 12.10%. HRMS (ESI) [M – Cl]^+^: *m*/*z* 543.0897, calculated for [C_22_H_14_N_5_^195^Pt]^+^, measured 543.0897. IR (ATR, cm^–1^): ν_max_ 469, 480, 685, 691, 710, 750, 770, 872, 920, 1082, 1101, 1188, 1206, 1302, 1385, 1449, 1468, 1489, 1535, 1578, 2887 w, 2972 w, 3050 w. UV–vis (CHCl_3_) λ_max_ (ε, M^–1^ cm^–1^): 302 (20,598), 377 (13,302), 406 (8113), 474 (14,583), 504 (15,799).

### Synthesis of Pt(**L**^**4**^)Cl

The same procedure as Pt(**L**^**3**^)Cl, except **HL**^**4**^ (0.035 g, 0.10 mmol), Pt(COD)Cl_2_ (0.042 g, 0.11 mmol), and DIPEA (19.5 μL, 0.11 mmol), was used. The solid was filtered, washed with MeOH, and dried to afford a dark red solid (0.041 g, 47%). ^1^H NMR (500 MHz, CDCl_3_): δ_H_ 10.45 (1H, dd, *J*_HH_ = 6.3, 1.7 Hz, CH), 10.04 (1H, d, *J*_HH_ = 7.2 Hz, CH), 9.38 (1H, d, *J*_HH_ = 9.1 Hz, CH), 8.30–8.33 (1H, m, CH), 8.14–8.18 (1H, m, CH), 7.99 (1H, ddd, *J*_HH_ = 8.1, 7.0, 1.7 Hz, CH), 7.87 (1H, ddd, *J*_HH_ = 8.0, 6.8, 1.3 Hz, CH), 7.80 (1H, d, *J*_HH_ = 8.0 Hz, CH), 7.67–7.77 (4H, m, CH), 7.30 (1H, d, *J*_HH_ = 7.3 Hz, CH), 7.11 (1H, ddd, *J*_HH_ = 7.0, 6.5, 1.8 Hz, CH). Elemental analysis: found, C 43.92%, H 1.78%, N 10.70%; calculated for C_22_H_14_N_5_PtCl.0.5CH_2_Cl_2_, C 43.49%, H 2.43%, N 11.27%. HRMS (ESI) [M + H]^+^: *m*/*z* 579.0664, calculated for [C_22_H_15_N_5_PtCl]^+^, measured 579.0659. IR (ATR, cm^–1^): ν_max_ 475, 540, 581, 685, 706, 743, 768, 781, 801, 918, 1084, 1094, 1109, 1123, 1198, 1387, 1466, 1493, 1533, 1580, 2889 w, 2980 w, 3055 w, 3123 w. UV–vis (CHCl_3_) λ_max_ (ε, M^–1^ cm^–1^): 277 (27,879), 363 (13,290), 392 (10,361), 489 (10,365), 515 (10,163).

### Synthesis of Pt(**L**^**5**^)Cl

The same procedure as Pt(**L**^**1**^)Cl, except **HL**^**5**^ (0.153 g, 0.5 mmol), Pt(COD)Cl_2_ (0.165 g, 0.4 mmol), and DIPEA (81.0 μL, 0.4 mmol), was used. The solid was filtered, washed with MeOH, and dissolved in toluene to afford a red solid (0.176 g, 68%). ^1^H NMR (500 MHz, CDCl_3_): δ_H_ 10.16 (2H, d, *J*_HH_ = 6.7 Hz, CH), 8.09 (2H, dd, *J*_HH_ = 5.5, 3.0 Hz, CH), 7.64 (2H, dd, *J*_HH_ = 5.5, 3.0 Hz, CH), 7.48 (2H, d, *J*_HH_ = 2.4 Hz, CH), 6.90 (2H, dd, *J*_HH_ = 6.7, 2.3 Hz, CH), 2.70 (4H, q, *J*_HH_ = 7.6 Hz, CH_2_), 1.34 (6H, t, *J*_HH_ = 7.6 Hz, CH_3_). ^13^C{^1^H} NMR (126 MHz, CDCl_3_): δ_C_: 155.8, 151.9, 151.2, 149.8, 137.7, 131.4, 126.2, 122.2, 120.3, 28.0, 13.6. Elemental analysis: found, C 45.58%, H 3.12%, N 11.84%; calculated for C_22_H_21_N_5_PtCl, C 45.17%, H 3.45%, N 11.97%. HRMS (ESI) [M + H]^+^: *m*/*z* 585.1133, calculated for [C_22_H_21_N_5_PtCl]^+^, measured 585.1146. IR (ATR, cm^–1^): ν_max_ 419, 465, 480, 698, 750, 781, 824, 833, 882, 895, 920, 1103, 1179, 1204, 1298, 1381, 1410, 1466, 1510, 1580, 1649, 1721, 2872 w, 2928 w, 2961 w, 3123 w. UV–vis (CHCl_3_) λ_max_ (ε, M^–1^ cm^–1^): 275 (17,475), 345 (9222), 385 (4706), 464 (5807), 490 (6993).

### Synthesis of Pt(**L**^**6**^)Cl

The same procedure as Pt(**L**^**1**^)Cl, except **HL**^**6**^ (0.056 g, 0.18 mmol), Pt(COD)Cl_2_ (0.074 g, 0.20 mmol), and DIPEA (34.4 μL, 0.20 mmol), was used. The solid was filtered, washed with MeOH, and dried to afford a dark red solid (0.034 g, 35%). ^1^H NMR (500 MHz, CDCl_3_): δ_H_ 9.33 (2H, d, *J*_HH_ = 4.4 Hz, CH), 8.15 (2H, dd = 5.5, 3.0 Hz, CH), 7.70 (2H, dd, *J*_HH_ = 5.5, 3.0 Hz, CH), 7.16 (2H, d, *J*_HH_ = 4.4 Hz, CH). Elemental analysis: found, C 31.80%, H 1.44%, N 12.49%; calcd for C_14_H_8_N_5_S_2_PtCl.0.75MeOH, C 31.36%, H 1.96%, N 12.40%. HRMS (ESI) [M + H]^+^: *m*/*z* 541.9637, calculated for [C_14_H_9_N_5_S_2_^196^PtCl]^+^, measured 541.9624. IR (ATR, cm^–1^): ν_max_ 527, 623, 644, 708, 741, 783, 876, 891, 930, 1074, 1109, 1165, 1175, 1194, 1209, 1238, 1294, 1319, 1385, 1466, 1508, 1541, 1601, 3082 w, 3127 w. UV–vis (CHCl_3_) λ_max_ (ε, M^–1^ cm^–1^): 262 (23,215), 296 (5385), 344 (8282), 362 (10,809), 383 (11,671), 406 (10,168), 477 (8579), 502 (7523).

## Results and Discussion

### Synthesis

The synthesis of BPI-type ligands is very well-known and traditionally follows one of the two routes from phthalonitrile: (i) Siegl’s method^39^ utilizes CaCl_2_, *n*-butanol, and heating with the amine; (ii) Linstead’s route^40^ generates 1,3-diiminoisoindoline, which is then reacted with the amine in ethanol. Both routes are effective for yielding symmetrical species. However, the reports of unsymmetrical BPI-type ligands, for example, where the heterocyclic donors are different, are extremely rare with previous attempts by Siegl being reported as unsuccessful.^39^ Kleeberg and Broring reported a method from 1,3-diiminoisoindoline where they were able to isolate a mixed thiazole/pyridine isoindoline ligand [and describe the resultant Pd(II) coordination chemistry] via a monosubstituted 1,3-diiminoisoindoline intermediate.^41^ The authors noted the rather problematic scrambling associated with the second condensation step that can lead to the competitive formation of symmetrical ligand species and is likely to limit the reported yields of the unsymmetrical target (<20%). In closely related work, the Pd(II) coordination chemistry of an unsymmetrical 1-(arylimino)-3-(2-hetarylimino)isoindolines has also been described where the ligand acts as a C^∧^N^∧^N pincer chelate via cyclopalladation.^42^

**Scheme 1:**
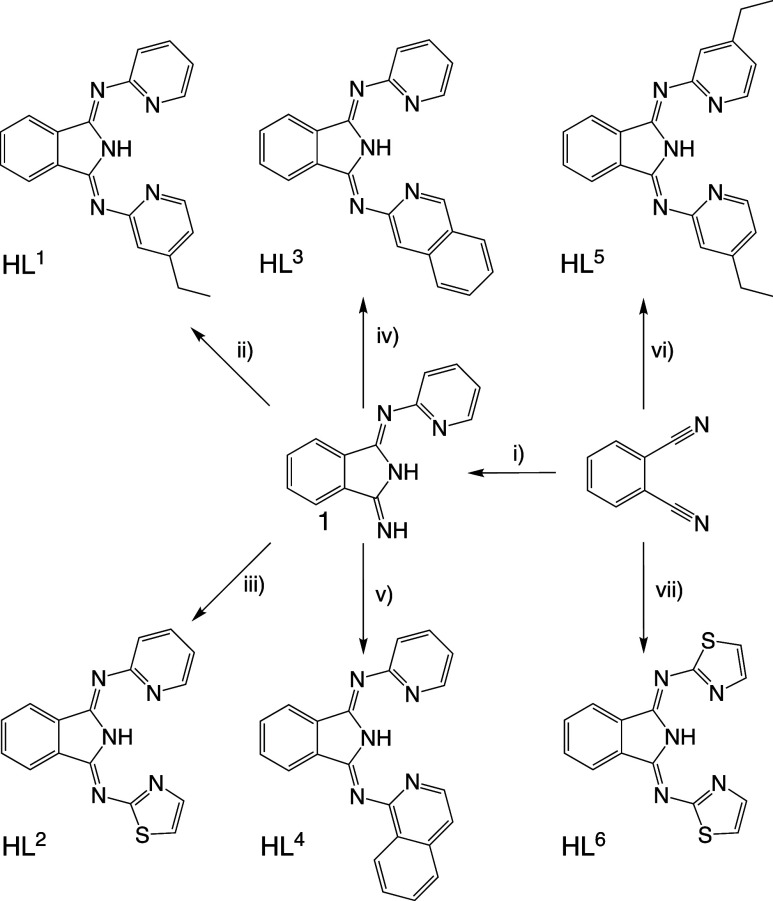
Synthetic Routes to the Family of Isoindoline-1,3-Diimine Ligands; Reagents: (i) 1 Equiv 2-Aminopyridine, NaH, THF; (ii) 1 Equiv 4-Ethyl-2-Aminopyridine, *n*-BuOH; (iii) 1 Equiv 2-Aminothiazole, *n*-BuOH; (iv) 1 Equiv 3-Aminoisoquinoline, *n*-BuOH; (v) 1-Aminoisoquinoline, *n*-BuOH; (vi) 2 Equiv 4-Ethyl-2-Aminopyridine, *n*-BuOH, CaCl_2_; and (vii) 2 Equiv 2-Aminothiazole, *n*-BuOH, CaCl_2_

With these prior observations in mind, our investigation focused on the synthesis of unsymmetrical BPI-type ligands for Pt(II). Our study showed that mixed-donor, unsymmetrical ligands (**HL**^**1**^**–HL**^**4**^) can be synthesized in a slightly different approach that required two steps and gave moderate-to-good overall yields (Scheme 1). First, 2-aminopyridine was treated with NaH in dry THF and then reacted with stoichiometric phthalonitrile to give the intermediate species *N*-(pyridine-2-yl)isoindoline-1,3-diimine (compound **1**, Scheme 1) which was isolated as a pale-yellow solid in good yield (75%). In its characterization, ^1^H NMR spectroscopy highlighted two unique N*H* resonances for this compound at 8.25 and 11.63 ppm (confirmed through loss of D/H exchange following the addition of D_2_O to the NMR sample–see Figure S1). The intermediate species (**1**) was then reacted further with a selection of heterocyclic amines (2-amino-4-ethylpyridine, 2-aminothiazole, 3-aminoisoquinoline, and 1-aminoisoquinoline) by heating in *n*-BuOH for 48 h to yield **HL**^**1**^**–HL**^**4**^, respectively. All ligands were purified using flash column chromatography and typically isolated as air-stable powders that possessed a yellow-to-gold coloration. Two further symmetrical ligands (**HL**^**5**^ and **HL**^**6**^) were isolated in a single step using Siegl’s method from phthalonitrile where CaCl_2_ was employed as a Lewis acid catalyst for the reaction (see Scheme 1).

**Scheme 2:**
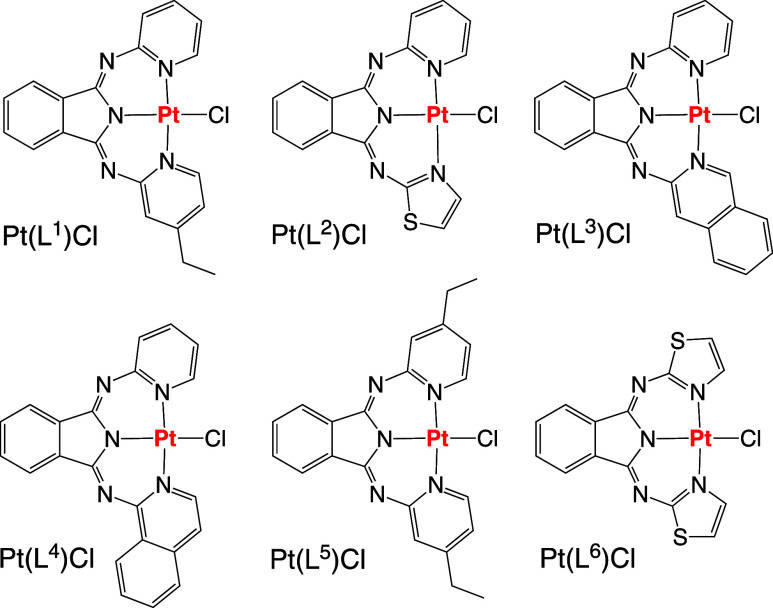
Structures of the Synthesized Pt(II) Complexes

The 1,3-diiminoisoindoline ligands were characterized using a typical array of methods and techniques. First, ^1^H NMR spectral analysis confirmed the formation of the desired ligands through a signature downfield resonance ca. 14 ppm which was attributed to isoindoline N*H*. In the case of the symmetrical ligands, the aromatic region of the spectra was relatively simple and, thus, indicative of the correct species. For **HL**^**5**^, additional triplet and quartet signals were noted at 1–3 ppm consistent with the presence of the ethyl substituents. The spectra of the unsymmetrical ligands gave well-resolved peaks in the aromatic region (see Figures S5, S9, S13, and S17) consistent with the integration of two different heterocyclic donors to the isoindoline core. The data also confirmed the absence of symmetrical byproducts that may result from scrambling during the final condensation step. ^13^C{^1^H} NMR spectra were also obtained for the ligands, wherein the furthest downfield signal(s) was typically associated with the 2-position of the imine substituent. For example, in the case of **HL**^**2**^, the C2 position of the thiazole ring appears at 172.4 ppm; the corresponding C2 position of the pyridine donor appears around 160 ppm. High-resolution mass spectrometry (HRMS) was obtained for each ligand with the [M + H]^+^ ion observed and in one case an *m*/*z* value consistent with a double-protonated species, [M + 2H]^2+^. IR spectra (solid samples) of the ligands revealed the N–H stretching frequency as a broad signal around 3200–3280 cm^–1^; additional strong bands at ca. 1550–1650 cm^–1^ likely include the C=N stretching frequency and the N–H bending mode (Figures S8, S12, S16, and S20).

The Pt(II) complexes (Scheme 2) were synthesized according to an adaption of a previous report^14^ whereby stoichiometric **HL**^***n***^, DIPEA, and Pt(COD)Cl_2_^43^ were heated to reflux in methanol for 24 h. During this time, the color of the reaction solution rapidly changed from yellow to orange/red depending upon the specific ligand. Upon cooling, the product precipitated from solution to afford air-stable, highly colored solids. Despite the limiting solubility in some cases, evidence for the successful complexation of the ligands was provided by NMR spectroscopy. First, the ^1^H NMR data (Figures S23, S27, S30, S33, S36, and S41) showed the absence of the N*H* resonance observed for the free ligands (ca. 14 ppm) indicative of coordination as the deprotonated isoindolate. Second, the aromatic resonances of the ligands were generally shifted downfield upon coordination to Pt(II). For example, in the symmetrical species Pt(**L**^**5**^)Cl, the furthest downfield resonance was observed at 10.16 ppm and attributed to the C6 position of the pyridinyl donors. This type of resonance was observed in all other examples; in the case of the quinoline species, Pt(**L**^**3**^)Cl and Pt(**L**^**4**^)Cl, an additional downfield signal was noted at 11.15 and 10.45 ppm, respectively, which again is attributed to the deshielded proton adjacent to the coordinated N atom. The aliphatic signals of the ethyl group within Pt(**L**^**1**^)Cl also demonstrated a subtle downfield shift upon coordination to Pt(II). Coupling to ^195^Pt (*I* = 1/2, 33.8%) was not well resolved in any of the ^1^H NMR spectra of the complexes, although some evidence of broadening on the bases of the peaks was noted. Where solubility allowed, ^13^C NMR spectra (Figures S24 and S37) were also recorded and confirmed the presence of the coordinated ligand and the correct number of carbon environments. HRMS data was recorded for the neutral complexes and typically showed evidence for the [M + H]^+^ ion and an adduct where MeCN had substituted the chloride ligand (thus giving a cationic fragment) during ionization.

**Figure 3:**
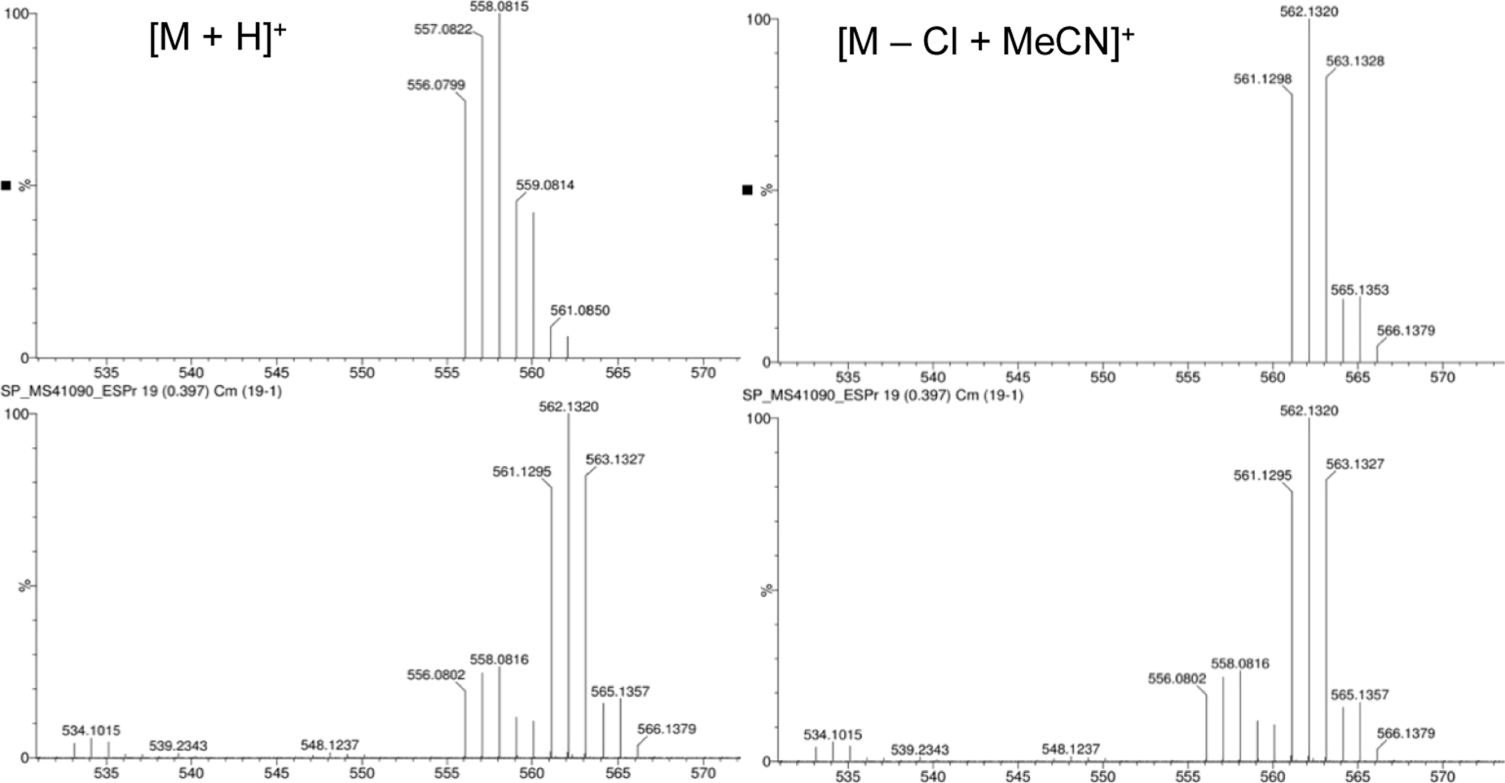
Example of the HRMS data for Pt(**L**^**1**^)Cl. Theoretical isotopic distributions (top) for [M + H]^+^ and [M – Cl + MeCN]^+^, with experimental data shown below.

Typically, the clusters of peaks associated with these species would overlap in the spectrum but with different isotopic distributions in each case (Figures 3, S25, S28, S31, S34, S38, S39, and S42), indicative of the presence or absence of ^35/37^Cl. Solid-state IR spectra of the complexes clearly highlighted the loss of the N–H stretching frequency ca. 3275 cm^–1^ which is consistent with deprotonation and subsequent coordination of the isoindolate fragment. Other ligand-based vibrational frequencies were clearly evident within the fingerprint region.

### X-ray Crystal Structure of Pt(L^5^)Cl

**Figure 4:**
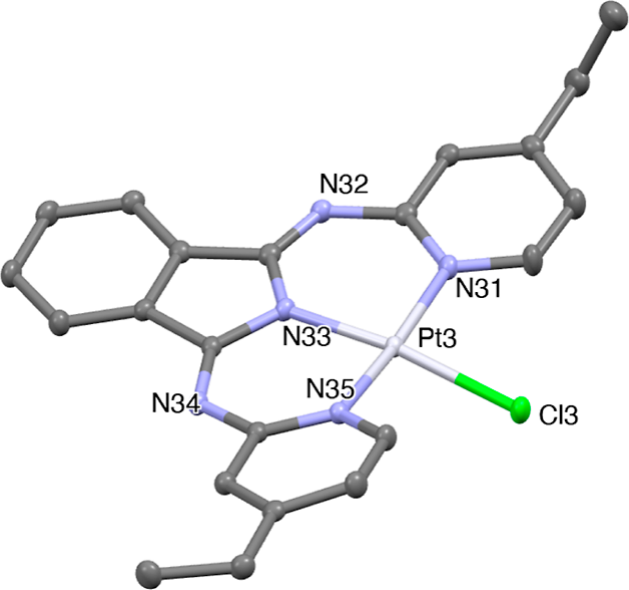
Structural representation obtained from single-crystal diffraction studies of Pt(**L**^**5**^)Cl showing one of the independent molecules of the asymmetric unit (*Z*′ = 2). Ellipsoids are drawn at 50%, and hydrogen atoms are not displayed.

Single crystals (orange, rod-shaped) were successfully isolated for Pt(**L**^**5**^)Cl following slow vapor diffusion of hexane into a chloroform solution of the complex. The structure was solved in the *P*1̅ (#2) space group and revealed two independent molecules in the asymmetric unit, with significant differences noted in the orientations of the ethyl groups. The obtained structure (Figure 4) revealed the anticipated coordination arrangement for the complex with a distorted square planar geometry evidently imposed by the ligands. It is notable that the Pt atom lies just out of the plane formed by the three nitrogen donors: Pt1 to N plane = 0.1654(13) Å and Pt3 to N plane = 0.1239(14) Å. The steric requirements of **L**^**5**^ impinge upon the auxiliary chloride which forces the Pt–Cl bond (N–Pt–Cl angle is around 170°) out of the plane defined by ‘Pt(**L**^**5**^)’, where Cl1 to N plane = 0.795(4) Å and Cl3 to N plane = 0.644(4) Å. The intramolecular H···Cl distances that result from this distortion are ca. 2.4–2.9 Å. The coordination bond lengths and angles are closely comparable to the previous report on Pt(BPI)Cl and reveal that the Pt–N(indolate) distance is the shortest of the Pt–N bonds (Table 1). Interestingly, the structure also revealed the absence of intermolecular Pt···Pt contacts (see the packing diagram, Figure S44).

**Table 1:** Key Structural Parameters That Describe the Coordination Sphere of Pt(**L**^**5**^)Cl

bond lengths (Å)					
Pt1	Cl1	2.3272(5)	Pt3	Cl3	2.3383(4)
Pt1	N1	2.0491(17)	Pt3	N31	2.0546(16)
Pt1	N3	1.9696(15)	Pt3	N33	1.9599(15)
Pt1	N5	2.0418(17)	Pt3	N35	2.0379(16)

bond angles (deg)							
N1	Pt1	Cl1	91.70(4)	N31	Pt3	Cl3	92.36(4)
N3	Pt1	Cl1	169.10(5)	N33	Pt3	Cl3	170.63(5)
N3	Pt1	N1	89.96(6)	N33	Pt3	N31	89.78(6)
N3	Pt1	N5	89.30(7)	N33	Pt3	N35	89.15(6)
N5	Pt1	Cl1	90.78(5)	N35	Pt3	Cl3	89.80(4)
N5	Pt1	N1	170.73(7)	N35	Pt3	N31	173.03(6)

### Electrochemistry

**Figure 5:**
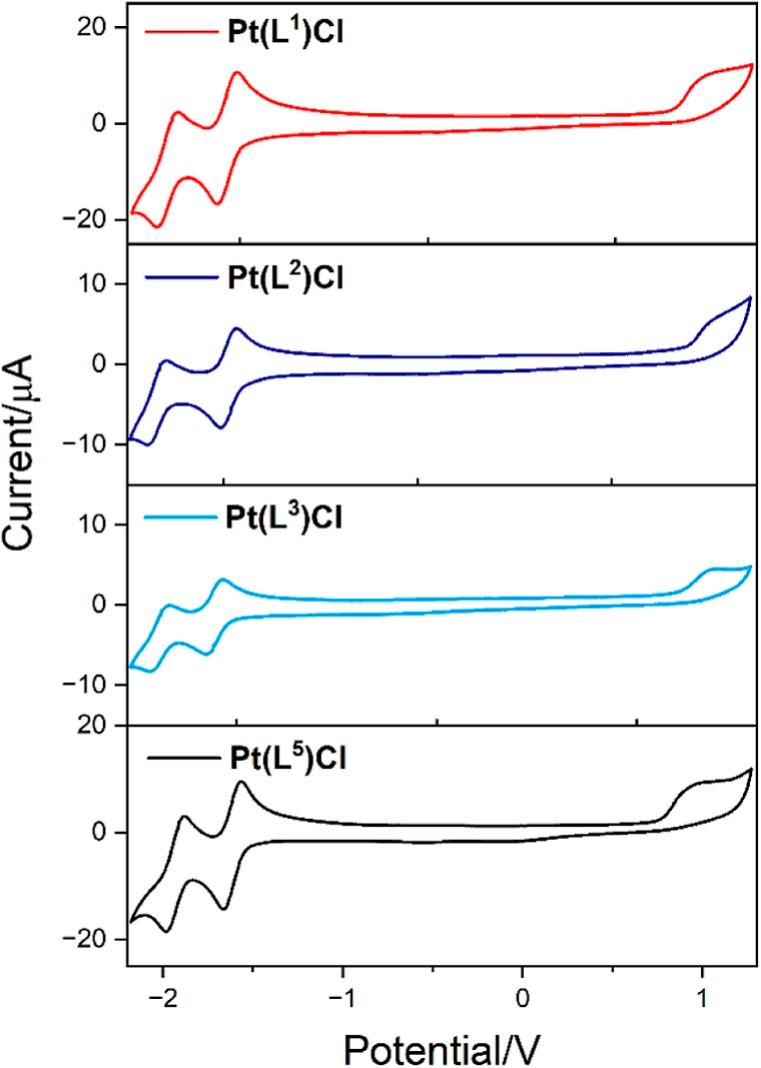
Examples of cyclic voltammograms of Pt(**L**^**1–3**^)Cl and Pt(**L**^**5**^)Cl. Measurements were recorded in degassed CH_2_Cl_2_, 293 K and 0.25 M [*n*-Bu_4_N][PF_6_] at a scan rate of 150 mV/s, relative to Fc/Fc^+^ (0 V).,

The electrochemical properties of the family of neutral platinum complexes were explored using cyclic voltammetry in CH_2_Cl_2_ solution at 1 mM concentration using [^n^Bu_4_N][PF_6_] as the supporting electrolyte (0.25 M) and the Fc/Fc^+^ redox couple as a reference (Table 2). Other than Pt(**L**^**6**^)Cl, all complexes exhibited an irreversible oxidation potential at +0.75 to +1.01 V which has been noted in related compounds. If the oxidation is metal centered, the irreversibility may be attributed to the resultant Pt(III) species which are known to be very unstable and prone to further reaction(s).^44^ Four complexes, Pt(**L**^**1–3**^)Cl and Pt(**L**^**5**^)Cl showed two fully reversible features (Figure 5) in the reduction window which are broadly comparable to those previously reported for Pt(BPI)Cl and related complexes.^13,14^ Comparison of Pt(**L**^**1**^)Cl with Pt(**L**^**5**^)Cl reveals that the first reduction becomes slightly harder for the latter complex, which is consistent with the presence of the additional electron-donating ethyl substituent on the pyridine donor. Interestingly, for Pt(**L**^**3**^)Cl, the first reduction is even more negative, which is perhaps surprising given the extended conjugation of the isoquinoline moiety. In stark contrast, isomeric Pt(**L**^**4**^)Cl showed reduction potentials that appeared to be irreversible, suggesting poor electrochemical stability for this particular species. The *bis*-thiazolo derivative, Pt(**L**^**6**^)Cl, was noted as the easiest to reduce, indicating that replacing pyridine donors with thiazole moieties raises the ligand-based reduction potential by ca. 0.1 V per thiazole [cf. Pt(**L**^**1**^)Cl and Pt(**L**^**2**^)Cl].^13^

**Table 2:** Electrode Oxidation and Reduction Potentials Obtained from the Cyclic Voltammetry of the Series of Pt(II) Complexes^a^

complex	*E*_red2_	*E*_red1_	*E*_ox_
Pt(**L**^**1**^)Cl	–1.89^b^	–1.57^b^	1.01
Pt(**L**^**2**^)Cl	–1.85^b^	–1.47^b^	1.01
Pt(**L**^**3**^)Cl	–1.89^b^	–1.61^b^	0.90
Pt(**L**^**4**^)Cl	–1.78^c^	–1.44^c^	0.75
Pt(**L**^**5**^)Cl	–1.91^b^	–1.60^b^	1.01
Pt(**L**^**6**^)Cl	–1.79^c^	–1.37^b^	-d

a Electrochemical potentials reported in volts (V) relative to Fc/Fc^+^ (0 V). Measurements were recorded in degassed CH_2_Cl_2_, 293 K, and 0.25 M [*n*-Bu_4_N][PF_6_] at a scan rate of 150 mV/s. All observed oxidation waves were irreversible.

b *E*_1/2_ values from reversible waves.

c Irreversible.

d Not observed.

### Spectroscopic Properties

**Figure 6:**
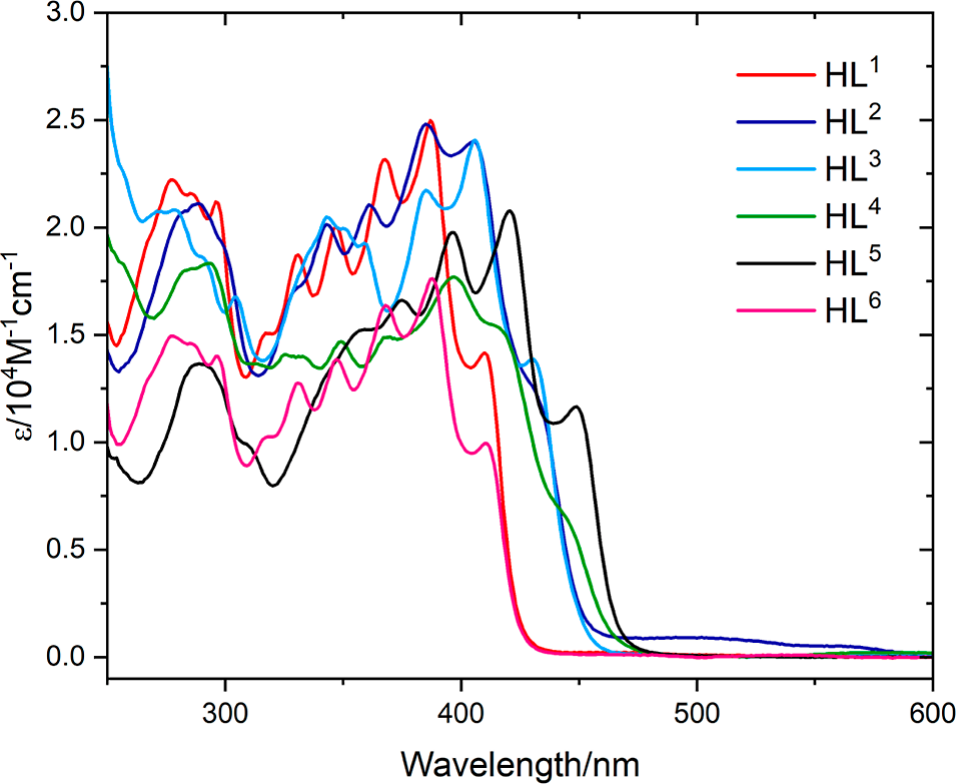
UV–vis spectra of the free ligands, **HL**^**1–6**^ (10^–5^ M in CHCl_3_).

A combination of experimental and theoretical approaches was used to probe the electronic behavior of the complexes. First, the UV–vis absorption data for the ligands and complexes was obtained in aerated CHCl_3_ and is presented in Table 2 and Figure 6. It is noteworthy that the spectrum for the intermediate species (**1**) revealed a lowest energy wavelength band at 373 nm; upon formation of the ligands, the lowest energy band is bathochromically shifted to 443 nm depending upon the nature of the heterocycle(s) and extent of increased conjugation. The ligands show a variety of strong absorption bands with peak maxima between 260 and 430 nm which are mainly attributed to different π → π* transitions associated with the various aromatic fragments, as noted previously. The lowest-energy spectral bands are characterized by very pronounced vibronic features indicative of a rigid structure.

**Figure 7:**
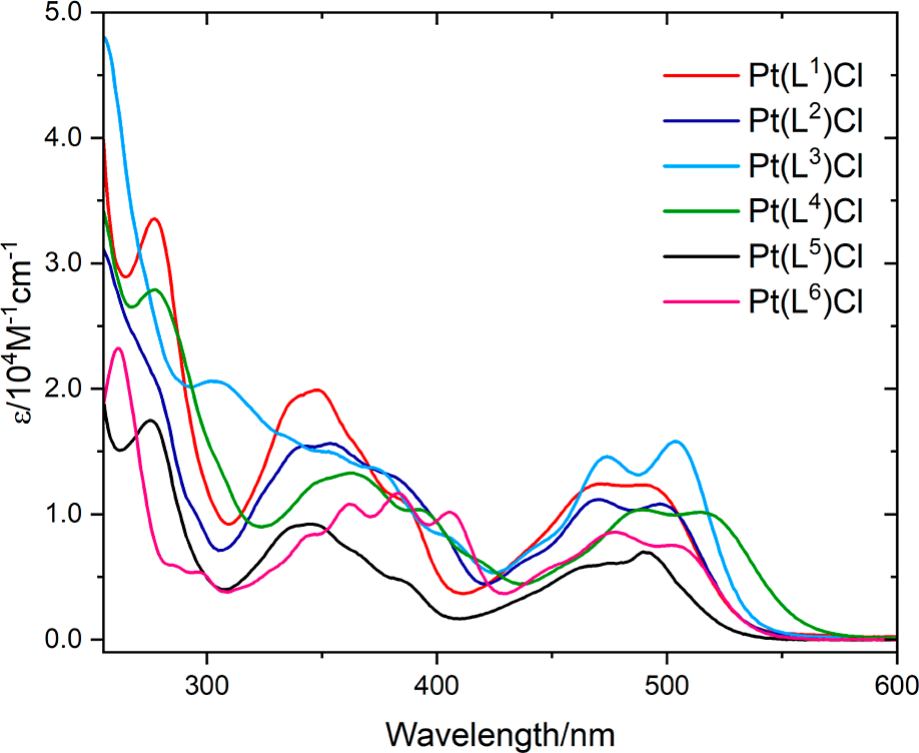
UV–vis spectra of the complexes Pt(**L**^**1–6**^)Cl (10^–5^ M in CHCl_3_).

Upon complexation with Pt(II), the expected change in the appearance of the spectra was observed. The spectra of the complexes are a composite of ligand-based transitions which are perturbed upon coordination to Pt(II) and an additional new feature that is strongly absorbing in the visible region with maxima peaking 491–515 nm; the isoquinoline derivative Pt(**L**^**4**^)Cl demonstrated the largest bathochromic shift within the series. Due to the intensity and subtle variation across the series, the visible band can be assigned as a spin-allowed transition, which is likely to have significant metal-to-ligand charge-transfer (^1^MLCT) band character, i.e., Pt(5d) → L(π*) (Figure 7). Further insight on this absorption feature was provided by DFT calculations.

Calculations were performed on the Pt(II) complexes to support the discussion and assignments of the features in the absorption spectra by using time-dependent DFT (TD-DFT) calculations and molecular orbital decomposition analysis. The calculated optimized geometries for the complexes were in excellent agreement with experimental structural data obtained previously for Pt(BPI)Cl^14^ and for Pt(**L**^**5**^)Cl described within this paper; bond lengths, angles, and apparent distortions are very well replicated by the calculations. It is noteworthy, therefore, that the calculated structure for Pt(**L**^**6**^)Cl is predicted to be much more planar than those of the other variants. Presumably, the intramolecular steric clashes between the auxiliary chloride ligand and the hydrogen substituents of the heterocycle may be slightly reduced in the case of the five-membered thiazole donors.

**Figure 8:**
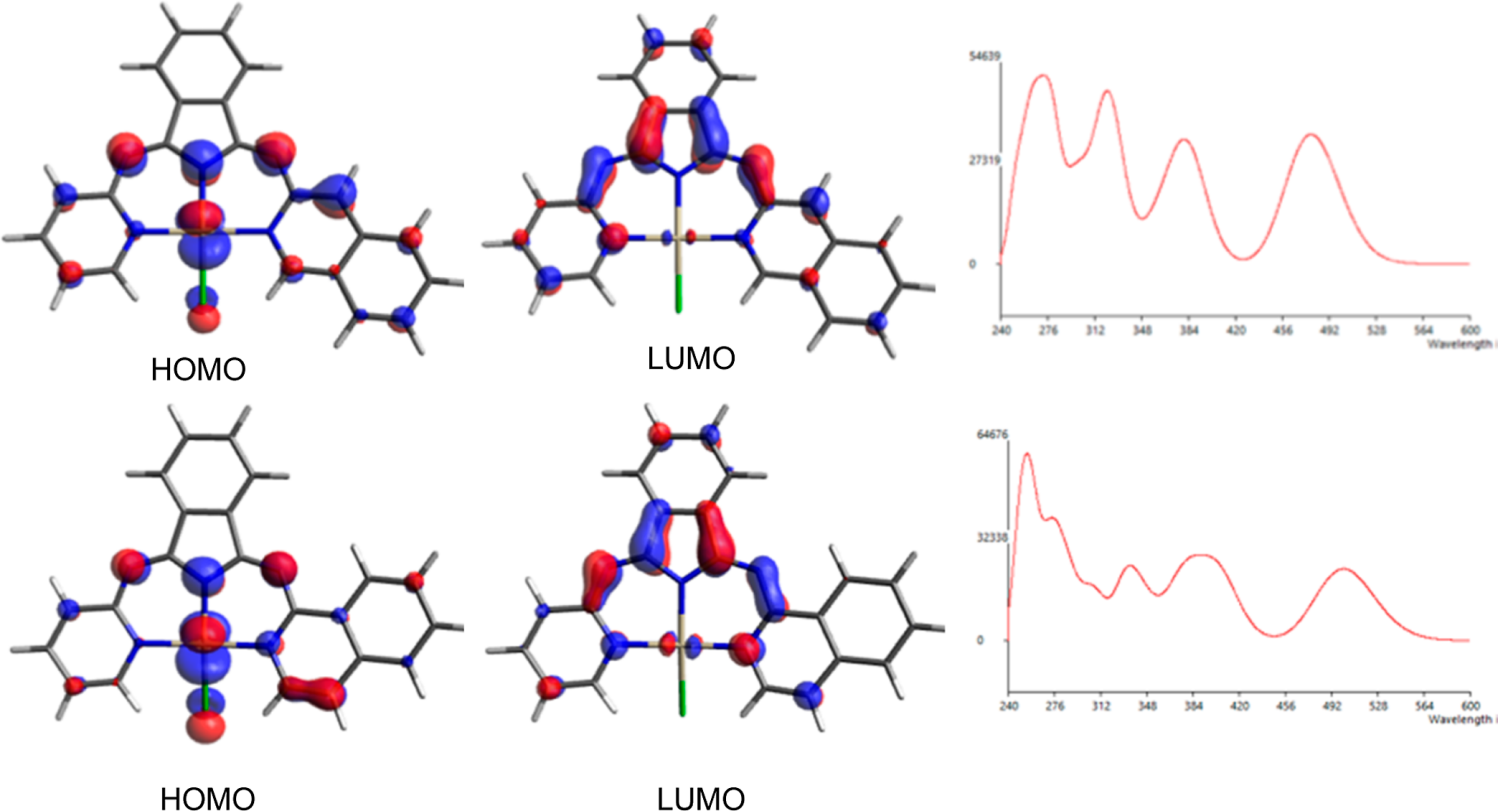
Pictorial representation of the Frontier orbitals for Pt(**L**^**3**^)Cl (top) and Pt(**L**^**4**^)Cl (bottom) and the DFT-calculated absorption spectra.

Overall, the calculations predict that the highest occupied molecular orbital (HOMO) is composed of both Pt (5d) and ligand-based orbitals. The Pt accounts for about 19–28%, the BPI-like ligand is ca. 60–75%, and the remainder (up to 11%) is associated with the chloride auxiliary ligand. The lowest unoccupied molecular orbital (LUMO) is predicted to be localized across the BPI ligand, with the heterocyclic substituents contributing significantly (Figure 8). Across the series, DFT calculations predicted that Pt(**L**^**4**^)Cl and Pt(**L**^**6**^)Cl may have the lowest-energy LUMOs; this was corroborated electrochemically, where both complexes were the most easily reduced. It is perhaps noteworthy that the high degree of structural planarity (defined by the Cl to N plane defined by ‘Pt(L^*n*^)’) predicted for Pt(**L**^**6**^)Cl may result in a slight reduction of both the HOMO and LUMO energies (Table 3).

**Table 3:** Calculated Energies of the Frontier Orbitals and Predictions of Selected Spectral and Structural Features for the Series of Pt(II) Complexes

complex	HOMO/eV	LUMO/eV	energy gap/eV	lowest energy absorption (*S*_0_ → *S*_1_)	Cl to Pt(L) plane/Å
**Pt(L**^**1**^**)Cl**	–6.15	–2.72	3.43	476 nm (*f* = 0.1853)	0.146
**Pt(L**^**2**^**)Cl**	–6.11	–2.69	3.42	479 nm (*f* = 0.1864)	0.044
**Pt(L**^**3**^**)Cl**	–5.96	–2.64	3.32	478 nm (*f* = 0.3376)	0.156
**Pt(L**^**4**^**)Cl**	–6.12	–2.85	3.27	501 nm (*f* = 0.2227)	0.155
**Pt(L**^**5**^**)Cl**	–6.07	–2.64	3.43	474 nm (*f* = 0.2075)	0.137
**Pt(L**^**6**^**)Cl**	–6.27	–2.97	3.30	488 nm (*f* = 0.1770)	0.009

In each case, the lowest-energy absorption spin-allowed band (*S*_0_ → *S*_1_) was calculated to be dominated by the HOMO–LUMO character. An orbital decomposition analysis predicted the different contributions to the HOMO and LUMO and, as shown (Table 4), the HOMO is likely characterized by Pt (5d) and ligand-based orbitals that comprise all three moieties (indolate, imine, and heterocycle). The different contributions may vary between the complexes; Pt(**L**^**3**^)Cl is predicted to have a smaller Pt and larger heterocycle influence on the HOMO. In all cases, the auxiliary chloride ligand is predicted to have a minor contribution. The LUMO is likely to be localized on the indolate and heterocycle orbitals: the HOMO–LUMO transitions are thus predicted to contain both MLCT and ILCT contributions. The calculations reproduce the wavelength positioning of this band in the visible region very nicely and correctly predict that the absorption is most bathochromically shifted for Pt(**L**^**4**^)Cl.

**Table 4:** Calculated HOMO and LUMO (%) Decomposition Analysis for Pt(**L**^***n***^)Cl Where **L**^***n***^ is Partitioned into Three Main Components (Indolate, Imine, and Heterocycle)

orbital	Pt	indolate	imine	heterocycle	Cl
Pt(L^1^)Cl					
LUMO	3	46	14	37	0
HOMO	28	16	19	26	11
Pt(**L**^**2**^)Cl					
LUMO	3	47	14	36	0
HOMO	24	14	19	34	9
Pt(**L**^**3**^)Cl					
LUMO	2	46	13	39	0
HOMO	19	15	20	40	6
Pt(**L**^**4**^)Cl					
LUMO	3	44	12	41	0
HOMO	24	13	17	36	10
Pt(**L**^**5**^)Cl					
LUMO	3	47	14	36	0
HOMO	28	16	19	27	11
Pt(**L**^**6**^)Cl					
LUMO	4	47	14	35	0
HOMO	22	13	18	38	10

**Figure 9:**
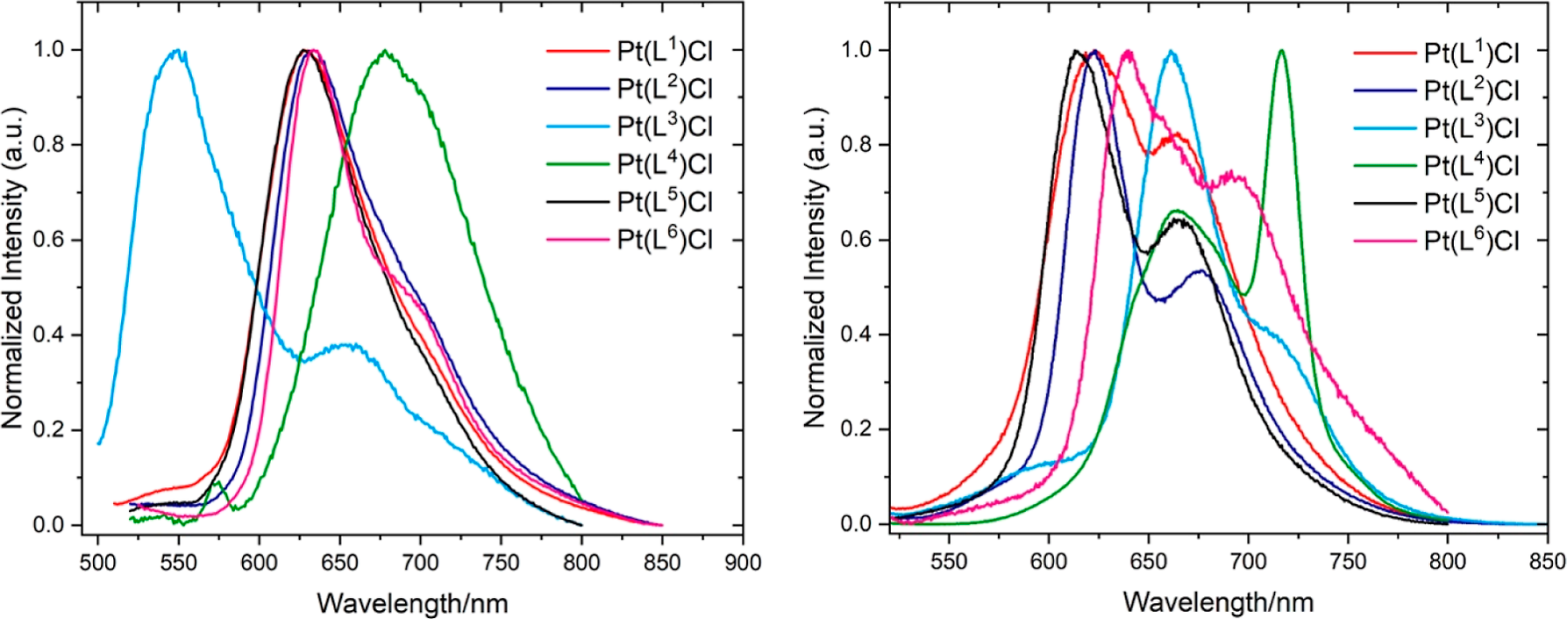
Normalized emission spectra for the family of Pt(II) complexes obtained in solution (left, 293 K, aerated CHCl_3_, 10^–5^ M; right, solid state).

The photophysical properties of the complexes were first measured in aerated fluid solution at room temperature. Due to the restricted solubility characteristics of the complexes, measurements were conducted in dilute chloroform to allow ease of comparison. Following excitation into the lowest-energy visible band, each of the complexes demonstrated photoluminescent character with peak maxima observed between 551 and 678 nm (Figure 9 and Table 5). Closely related Pt(**L**^**1**^)Cl and Pt(**L**^**5**^)Cl share the same emission maximum of 629 nm which is consistent with that reported by Chen and coworkers for the unsubstituted parent complex, Pt(BPI)Cl (λ_em_ = 631 nm). Our studies show that incorporating either one (Pt(**L**^**2**^)Cl) or two thiazole donors (Pt(**L**^**6**^)Cl) into the ligand induces very minor perturbations of the emission wavelengths at 631 and 633 nm, respectively. Integration of the isoquinoline induces more profound changes which depend upon the precise isomeric identity of the donor. For example, the 1-aminoisoquinoline donor in Pt(**L**^**4**^)Cl induces a larger bathochromic shift to 678 nm. The observation of the bathochromic shift is consistent with previous work on a symmetrical bis-isoquinoline Pt(II) complex reported by Thompson and coworkers (λ_em_ = 711 nm in deaerated toluene).^14^ Therefore, it appears that a progressive redshift in the emission can be achieved through the combination of different heterocyclic donors. Finally, the 3-aminoisoquinoline derivative, Pt(**L**^**3**^)Cl, showed quite different behavior in solution with weak emission described by two contributions to the emission profile at 551 and 661 nm. The latter shares characteristics with the other complexes in the series, while the 551 nm band is likely due to residual ligand-centered fluorescence as indicated by its short lifetime.

**Table 5:** Absorption and Photoluminescence Data for the Pt(II) Complexes^a^

complex	λ_abs_/nm^b^(ε × 10^4^/M^–^^1^ cm^–^^1^)	λ_em_/nm^c^	λ_em_ (solid)/nm^c^	τ/μs^d^	Φ_PL_ (%)^e^
**Pt(L**^**1**^**)Cl**	277 (3.4), 348 (2.0), 386 (1.1), 472 (1.2), 491 (1.2)	629	623, 664 sh	0.31 (1.08)	0.6 (0.8)
**Pt(L**^**2**^**)Cl**	341 (1.5), 354 (1.6), 383 (1.3), 471 (1.1) 497 (1.1)	631	623, 676 sh	0.27 (1.03)	0.4 (0.7)
**Pt(L**^**3**^**)Cl**	302 (2.1), 377 (1.3) 406 (0.8), 474 (1.5), 504 (1.6)	551, 653	662, 710 sh	<0.01	<0.1 (<0.1)
**Pt(L**^**4**^**)Cl**	277 (2.8), 363 (1.3), 392 (1.0), 489 (1.0), 515 (1.0)	678	663, 717	0.11 (0.18)	0.1 (0.2)
**Pt(L**^**5**^**)Cl**	275 (1.7), 345 (0.9), 385 (0.5), 464 (0.6), 490 (0.7)	629	615, 666 sh	0.32 (1.14)	0.6 (0.8)
**Pt(L**^**6**^**)Cl**	262 (2.3), 296 (0.5), 344 (0.8), 362 (1.1), 383 (1.2), 406 (1.0), 477 (0.9), 502 (0.8)	633	640, 694 sh	0.32 (2.67)	0.5 (2.0)

a All measurements obtained in CHCl_3_ at 293 K unless otherwise stated.

b 10^–5^ M.

c λ_ex_ = 475–500 nm.

d Observed lifetime, λ_ex_ = 295 nm; deoxygenated values shown in parentheses.

e Using [Ru(bipy)_3_][PF_6_]_2_ as a reference (Φ = 0.018 aerated; 0.095 degassed); deoxygenated values shown in parentheses.^28^

Low-temperature, total emission spectra were also obtained on frozen solutions at 77 K (Figure S45). Generally, the spectra showed a relative hypsochromic shift characteristic of CT-type emission from a rigid, frozen matrix. Again, Pt(**L**^**4**^)Cl displayed the deepest redshift in the series, while the spectrum of Pt(**L**^**3**^)Cl was dominated by a vibronically structured peak (470, 502, and 537 nm), which is again consistent with ligand-centered emission.

The complexes were also analyzed in the solid state, and each was shown to be emissive in the red-to-deep-red region with maxima between 623 and 717 nm (Figure 9); these emission wavelengths broadly tally with those observed in solution, implying that the solid-state spectra are not due to excimeric behavior supported by ligand π–π interactions.^45^ The spectra of Pt(**L**^***n***^)Cl (where *n* = 1, 2, 5, and 6) are comparable in appearance to a broad emission feature (peaking at 623–640 nm) with a lower-energy shoulder. In contrast to its solution-phase spectra, Pt(**L**^**3**^)Cl now showed an obvious emission maximum at ca. 662 nm (with a shoulder at 710 nm). The 551 nm band evident in the solution state (tentatively assigned to residual ligand fluorescence) is now strongly diminished, which may imply that potential triplet-state quenching pathways that result from the distorted coordination sphere of Pt(**L**^**3**^)Cl may be suppressed in the solid state. The spectrum of Pt(**L**^**4**^)Cl is also noteworthy, with a broad peak at 666 nm [closely matching Pt(**L**^**3**^)Cl], but a much more pronounced secondary feature at 716 nm, the positioning of which was independent of a range of excitation wavelengths, 480–525 nm. The solid-state data show that within the series of complexes, the emission maxima of the quinoline derivatives, Pt(**L**^**3**^)Cl and Pt(**L**^**4**^)Cl, are bathochromically shifted due to the additional conjugation of the quinoline donor.

With the exception of Pt(**L**^**3**^)Cl, time-resolved luminescence measurements revealed decay profiles that fitted well to a single-exponential function yielding lifetimes in the range of 111–315 ns under aerated conditions, which is consistent with previous work. Upon deoxygenation, the lifetime values were generally extended to the microsecond domain [for example, Pt(**L**^**5**^)Cl, was recorded as 1.14 μs, which compares favorably to the reported value of Pt(BPI)Cl of 0.97 μs in CH_2_Cl_2_^14^] and confirms the triplet nature of the excited state. Taken together, the spectral appearance and lifetime characteristics of the complexes generally imply that these are triplet emitters with significant charge-transfer character, which as supported by DFT is probably best described as an admixture of ^3^MLCT/^3^ILCT. The generally low emissivity of the complexes, and particularly Pt(**L**^**3**^)Cl, in solution perhaps demonstrates how sensitive this particular architecture can be to the nature and isomeric configuration of the heterocyclic donors that lead to distortions in the coordination sphere. Interestingly, Pt(**L**^**6**^)Cl appears to be the most emissive species (highest quantum yield and longest lifetime under deoxygenated conditions), and this may be due to the greater planarity (and thus reduced distortion) of the coordination sphere that is predicted for the bis(2-thiazolylimino)isoindolate variant.

## Conclusions

BPI-type ligands continue to attract significant attention in coordination chemistry disciplines due to their attractive chelating attributes and resultant functionality, and broad application of metal complexes. The current study has demonstrated a methodology for synthesizing unsymmetrical, mixed heterocyclic donor derivatives of the BPI core, yielding pincer-like species that readily coordinate to Pt(II) to give distorted square planar complexes. In this manner, different pyridyl, thiazole, and quinoline donors can be incorporated into a ligand architecture providing opportunities for tuning the donor ability and steric imposition of the ligands at the metal center. The complexes are redox active: while the irreversible oxidation features are typical of Pt(II) species, most of the complexes showed (quasi) reversible reduction features that are ligand based. All of the complexes were photoluminescent following excitation into a visible absorption band that is likely described by an admixture of MLCT and ILCT character, as inferred from computational studies. The complexes are generally revealed to be triplet emitters, with lifetimes extended to the microsecond domain in a deoxygenated solvent. The different combinations of heterocyclic donors that were investigated in this study show that the electronic properties of this class of complexes can be tuned. Integration of a 1-aminoisoquinoline donor provides a 50 nm bathochromic shift in the emission maximum relative to the other variants. Further studies could consider the investigation of these complexes as responsive vapochromic materials, as noted in other square planar Pt(II) complexes with Pt(N^∧^N^∧^N)Cl coordination spheres.^46^ As discussed earlier, the interesting applications evident for BPI metal complexes, including as catalysts, suggest that fine-tuning of ligand properties through the development of unsymmetrical BPI-type variants should provide a fruitful avenue for researchers in such fields of study.

## Supporting Information Available

^1^H, ^13^C NMR, and IR spectra for all compounds, data collection parameters for the X-ray crystal structure, and additional photophysical data (PDF)
