# Supplementary material for: Luminescent Pt(II) Complexes Using Unsymmetrical Bis(2-pyridylimino)isoindolate Analogues
Source: Inorg Chem. 2024 Apr 24;63(18):8273–85. doi: 10.1021/acs.inorgchem.4c00558 (PMC11080048; doi:10.1021/acs.inorgchem.4c00558)
Supplement: Supplementary file 1 — ic4c00558_si_001.pdf [file ic4c00558_si_001.pdf]

Supplementary Information

Luminescent Pt(II) Complexes Using Unsymmetrical bis(2-pyridylimino)isoindolate (BPI) Analogues

Ellie N. Payce,[a] Richard C. Knighton,[a] James A. Platts,[a] Peter N. Horton,[b] Simon J. Coles[b] and Simon J. A. Pope[a*]

[a]School of Chemistry, Main Building, Cardiff University, Cardiff CF10 3AT, Cymru/Wales; [b]UK National Crystallographic Service, Chemistry, Faculty of Natural and Environmental Sciences, University of Southampton, Highfield, Southampton, SO17 1BJ, England, UK. Email: popesj@cardiff.ac.uk

Contents

Figure S1	^{1}H NMR spectrum of **1** in $CDCl_3$ (500 MHz). Inset shows loss of NH peaks via D_2O exchange.	3
Figure S2	^{13}C NMR spectrum of **1** in $CDCl_3$ (500 MHz).	3
Figure S3	HRMS spectrum of **1**.	4
Figure S4	Infrared spectrum of **1**.	4
Figure S5	^{1}H NMR spectrum of **HL^{1}** in $CDCl_3$ (500 MHz).	5
Figure S6	^{13}C NMR spectrum of **HL^{1}** in $CDCl_3$ (500 MHz).	5
Figure S7	HRMS spectrum of **HL^{1}**.	6
Figure S8	Infrared spectrum of **HL^{1}**.	6
Figure S9	^{1}H NMR spectrum of **HL^{2}** in $CDCl_3$ (500 MHz). Inset shows aromatic region.	7
Figure S10	^{13}C NMR spectrum of **HL^{2}** in $CDCl_3$ (500 MHz).	7
Figure S11	HRMS spectrum of **HL^{2}**.	8
Figure S12	Infrared spectrum of **HL^{2}**.	8
Figure S13	^{1}H NMR spectrum of **HL^{3}** in $CDCl_3$ (500 MHz). Inset shows aromatic region.	9
Figure S14	^{13}C NMR spectrum of **HL^{3}** in $CDCl_3$ (500 MHz).	9
Figure S15	HRMS spectrum of **HL^{3}**.	10
Figure S16	Infrared spectrum of **HL^{3}**.	10
Figure S17	^{1}H NMR spectrum of **HL^{4}** in $CDCl_3$ (500 MHz). Inset shows aromatic region.	11
Figure S18	^{13}C NMR spectrum of **HL^{4}** in $CDCl_3$ (500 MHz).	11
Figure S19	HRMS spectrum of **HL^{4}**.	12
Figure S20	Infrared spectrum of **HL^{4}**.	12
Figure S21	^{1}H NMR spectrum of **HL^{6}** in $CDCl_3$ (500 MHz).	13
Figure S22	^{13}C NMR spectrum of **HL^{6}** in $CDCl_3$ (500 MHz).	13
Figure S23	^{1}H NMR spectrum of Pt(**L^{1}**)Cl in $CDCl_3$ (500 MHz). Inset shows aromatic region.	14
Figure S24	^{13}C NMR spectrum of Pt(**L^{1}**)Cl in $CDCl_3$ (500 MHz).	14
Figure S25	HRMS spectrum of Pt(**L^{1}**)Cl.	15
Figure S26	Infrared spectrum of Pt(**L^{1}**)Cl.	15
Figure S27	^{1}H NMR spectrum of Pt(**L^{2}**)Cl in $CDCl_3$ (500 MHz). Inset shows aromatic region.	16
Figure S28	HRMS spectrum of Pt(**L^{2}**)Cl.	16
Figure S29	Infrared spectrum of Pt(**L^{2}**)Cl.	17

Figure S30	^{1}H NMR spectrum of Pt(**L**^{3})Cl in $CDCl_3$ (500 MHz). Inset shows aromatic region.	17
Figure S31	HRMS spectrum of Pt(**L**^{3})Cl.	18
Figure S32	Infrared spectrum of Pt(**L**^{3})Cl.	18
Figure S33	^{1}H NMR spectrum of Pt(**L**^{4})Cl in $CDCl_3$ (500 MHz). Inset shows aromatic region.	19
Figure S34	HRMS spectrum of Pt(**L**^{4})Cl.	19
Figure S35	Infrared spectrum of Pt(**L**^{4})Cl.	20
Figure S36	^{1}H NMR spectrum of Pt(**L**^{5})Cl in $CDCl_3$ (500 MHz). Inset shows aromatic region.	20
Figure S37	^{13}C NMR spectrum of Pt(**L**^{5})Cl in $CDCl_3$ (500 MHz).	21
Figure S38	HRMS spectrum of Pt(L^{5})Cl.	21
Figure S39	HRMS spectrum of Pt(L^{5})Cl.	22
Figure S40	Infrared spectrum of Pt(**L**^{5})Cl.	22
Figure S41	^{1}H NMR spectrum of Pt(**L**^{6})Cl in $CDCl_3$ (500 MHz).	23
Figure S42	HRMS spectrum of Pt(**L**^{6})Cl.	23
Figure S43	Infrared spectrum of Pt(**L**^{6})Cl.	24
Table S1	Data collection parameters for the X-ray crystal structure	25
Figure S44	Packing diagram for the X-ray crystal structure	
Figure S45	Total emission spectra recorded at 77 K of Pt(**L**^{n})Cl (4:1, chloroform/toluene).	26

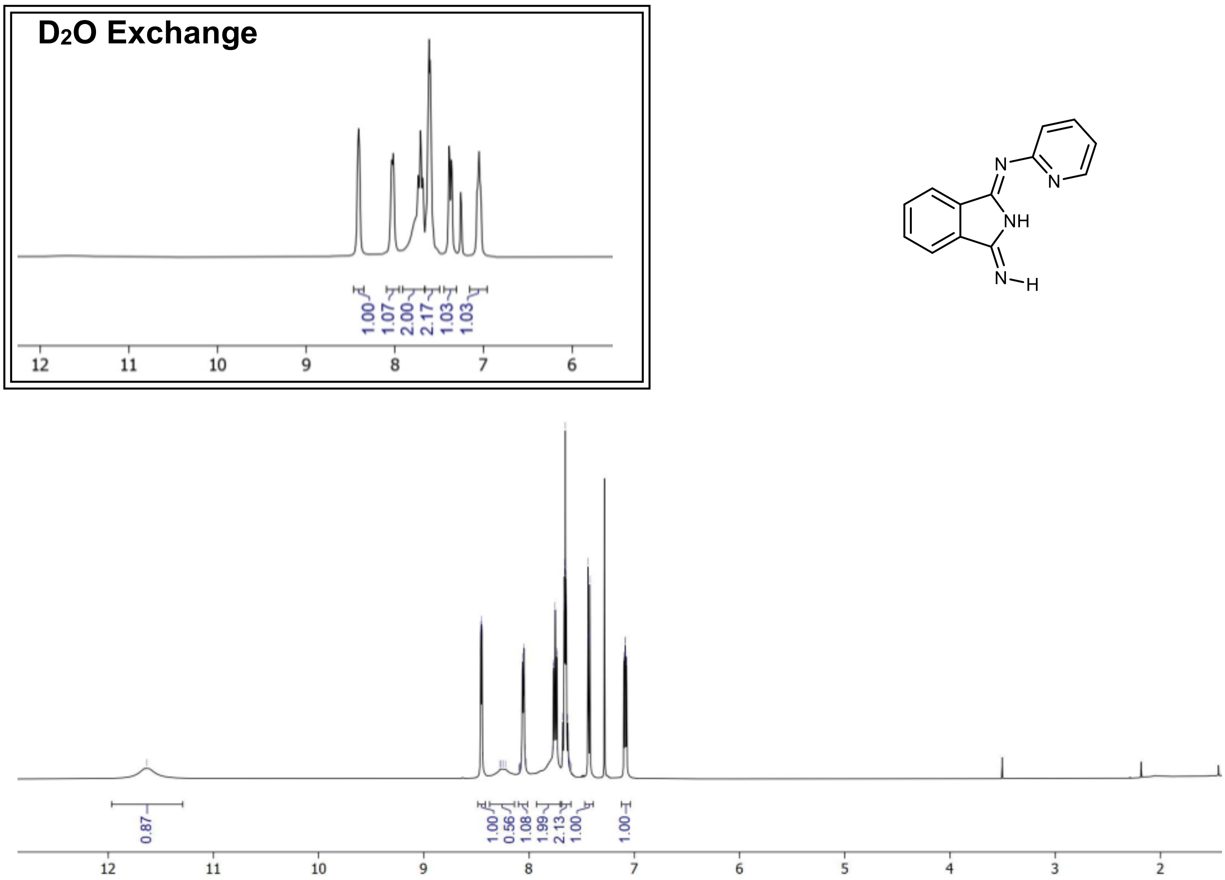

Figure S1. ^{1}H NMR spectrum of **1** in $CDCl_3$ (500 MHz). Inset shows loss of NH peaks via D_2O exchange.

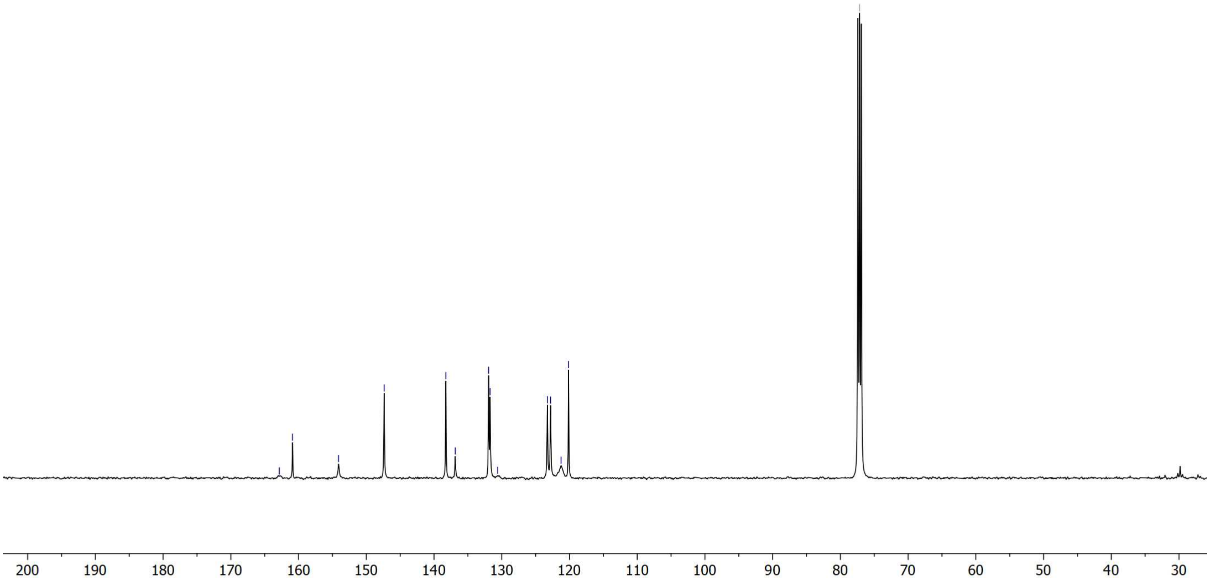

Figure S2. ^{13}C NMR spectrum of **1** in $CDCl_3$ (500 MHz).

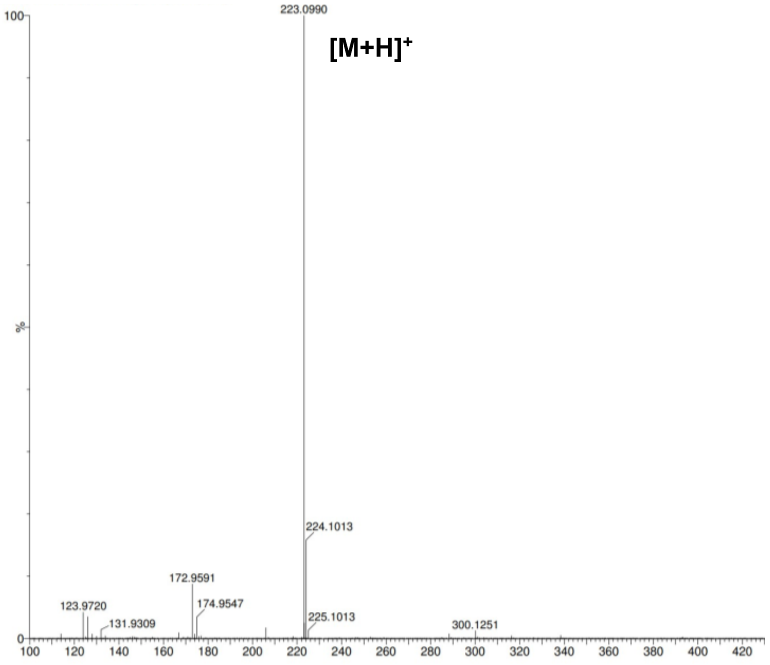

Figure S3. HRMS spectrum of **1**.

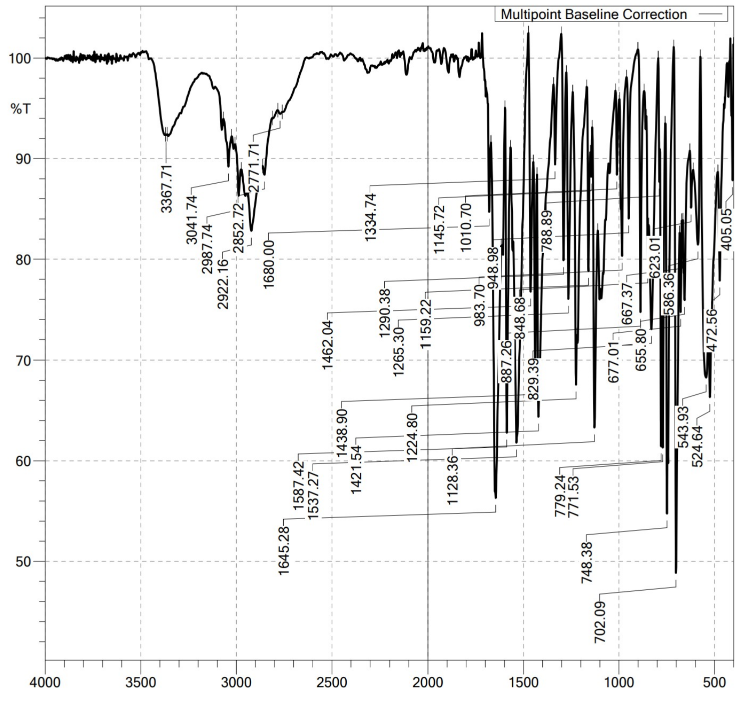

Figure S4. Infrared spectrum of **1**.

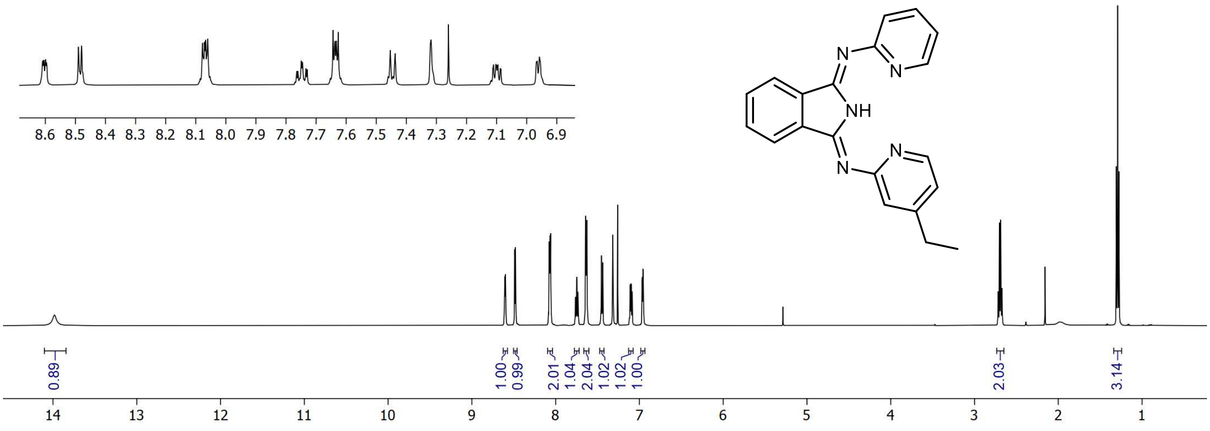

Figure S5. ^{1}H NMR spectrum of **HL[1]** in $CDCl_3$ (500 MHz).

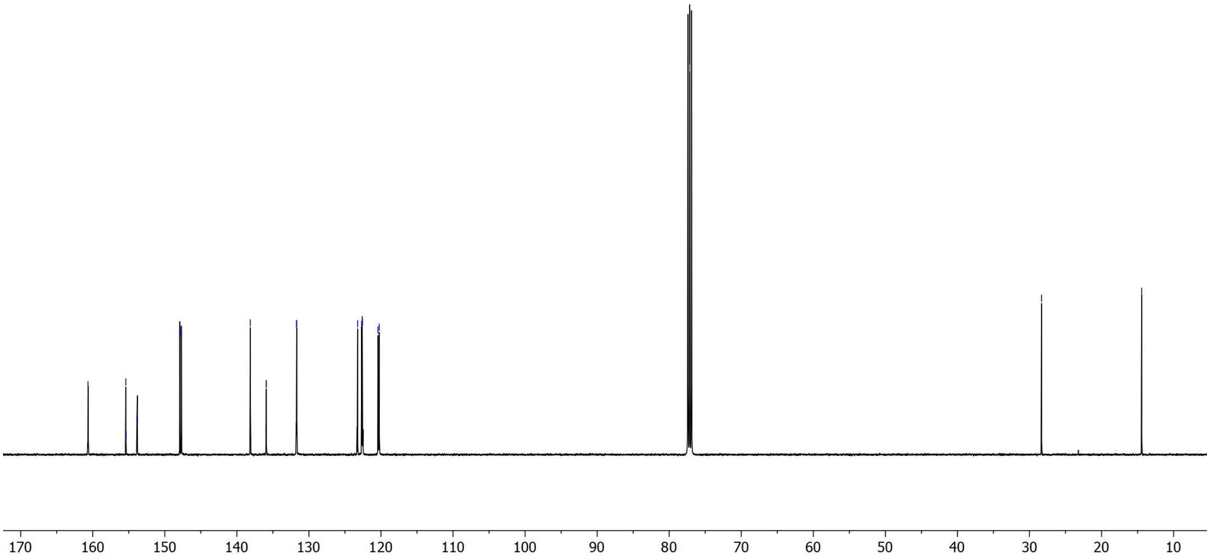

Figure S6. ^{13}C NMR spectrum of **HL[1]** in $CDCl_3$ (500 MHz).

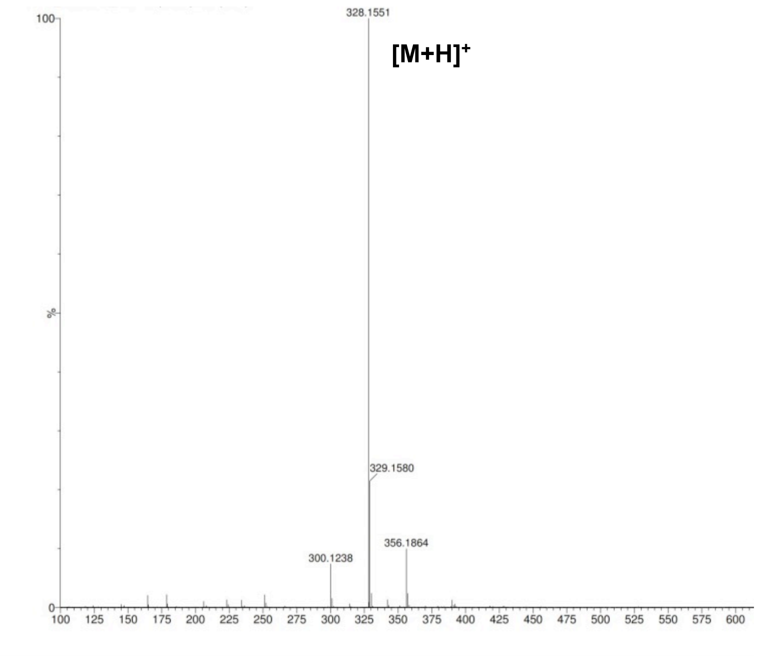

Figure S7. HRMS spectrum of **HL[1]**.

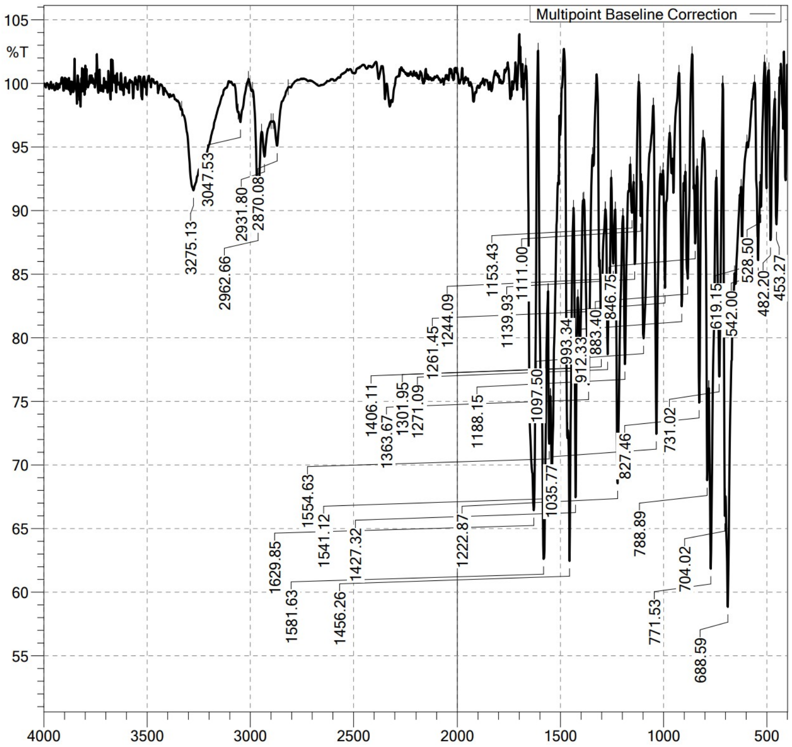

Figure S8. Infrared spectrum of **HL[1]**.

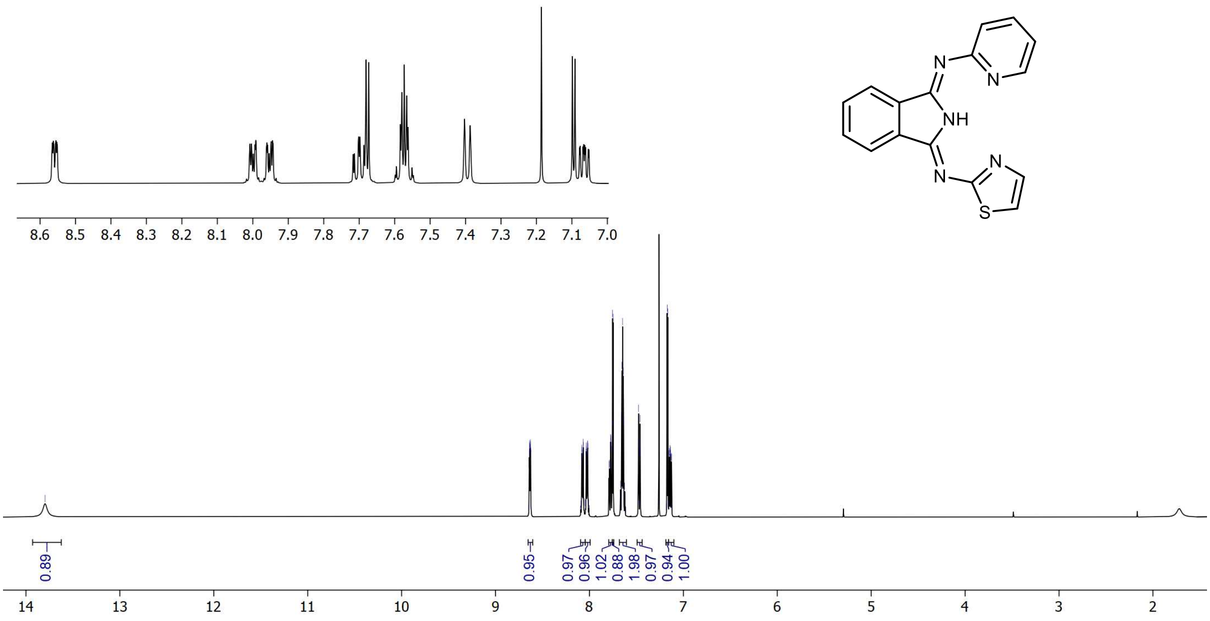

Figure S9. ^{1}H NMR spectrum of **HL^{2}** in $CDCl_3$ (500 MHz). Inset shows aromatic region.

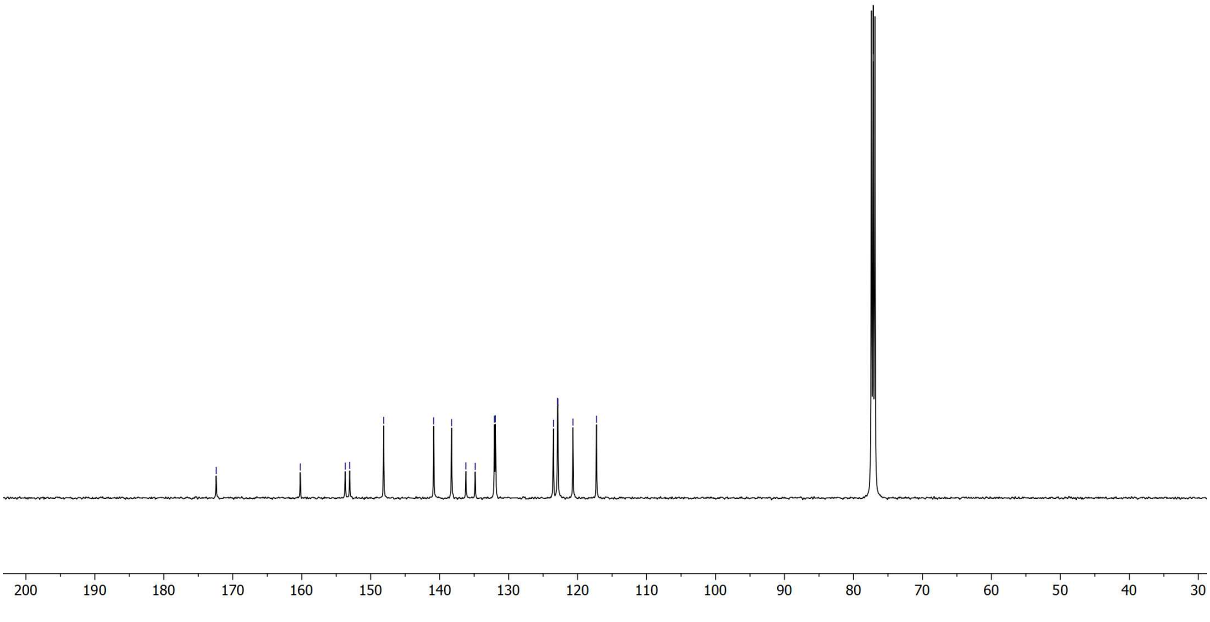

Figure S10. ^{13}C NMR spectrum of **HL^{2}** in $CDCl_3$ (500 MHz).

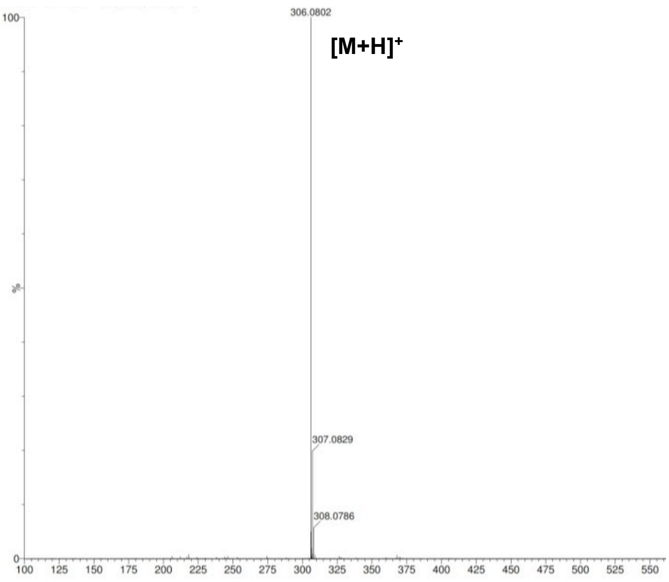

Figure S11. HRMS spectrum of $\mathbf{HL^2}$.

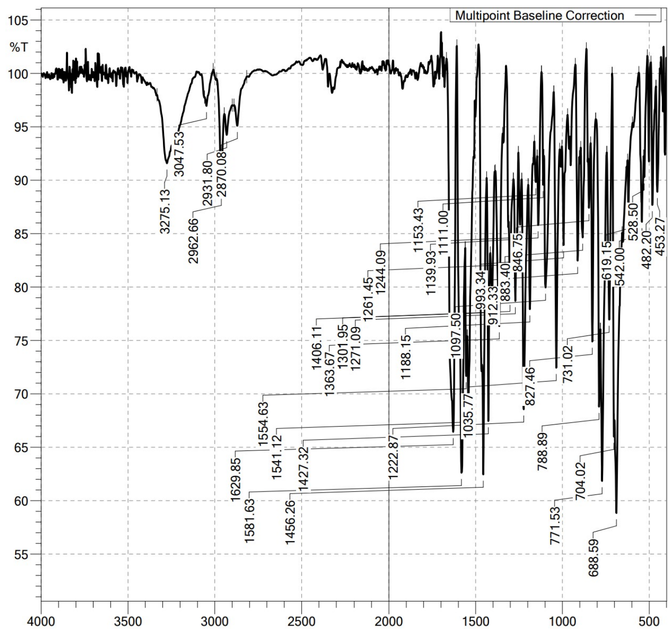

Figure S12. Infrared spectrum of $\mathbf{HL^2}$.

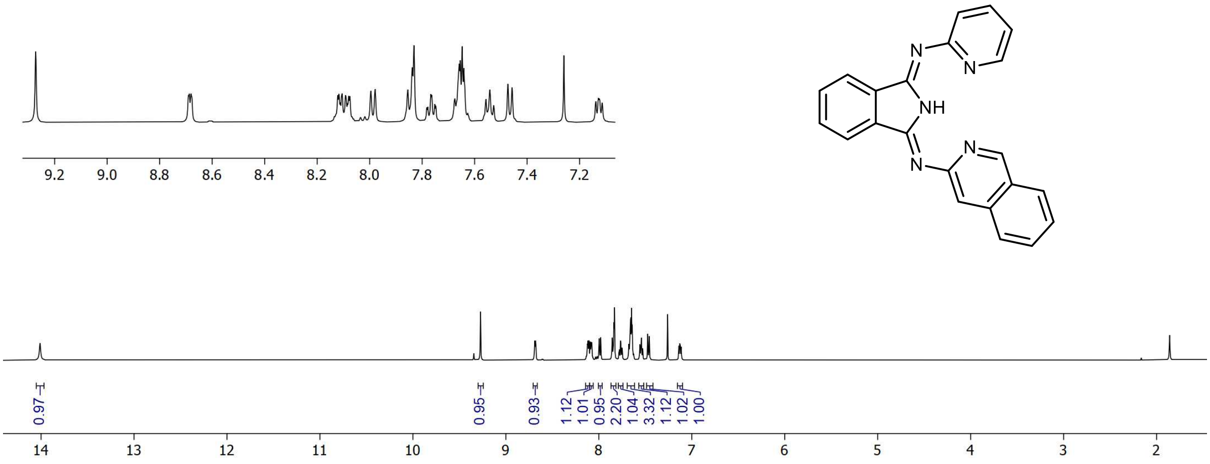

Figure S13. ^{1}H NMR spectrum of **HL^{3}** in $CDCl_3$ (500 MHz). Inset shows aromatic region.

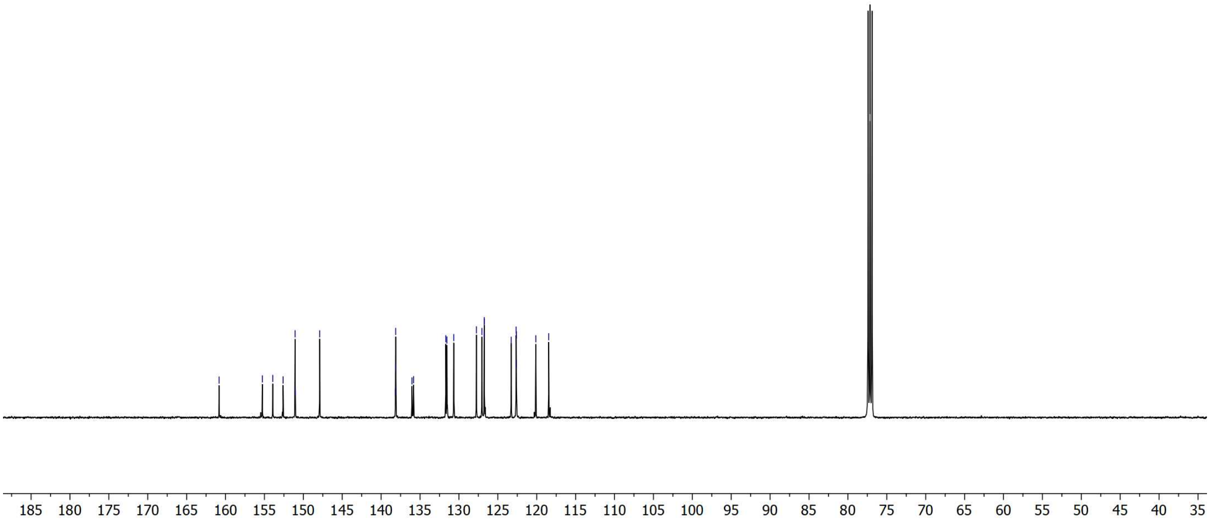

Figure S14. ^{13}C NMR spectrum of **HL^{3}** in $CDCl_3$ (500 MHz).

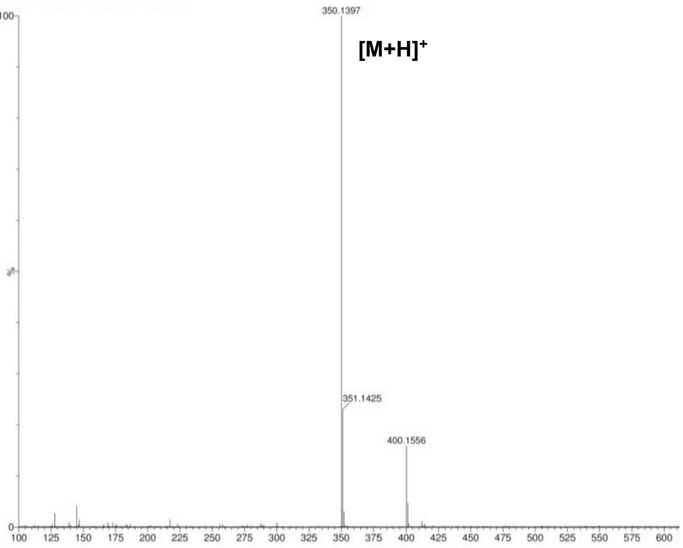

Figure S15. HRMS spectrum of **HL^3**.

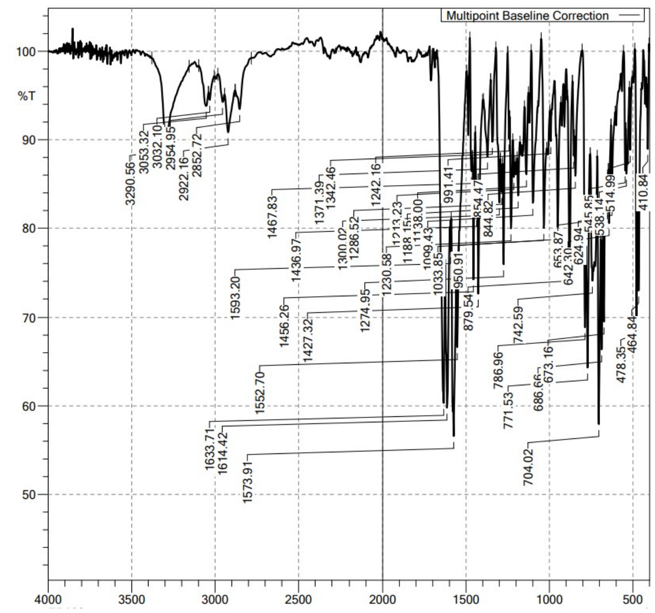

Figure S16. Infrared spectrum of **HL^3**.

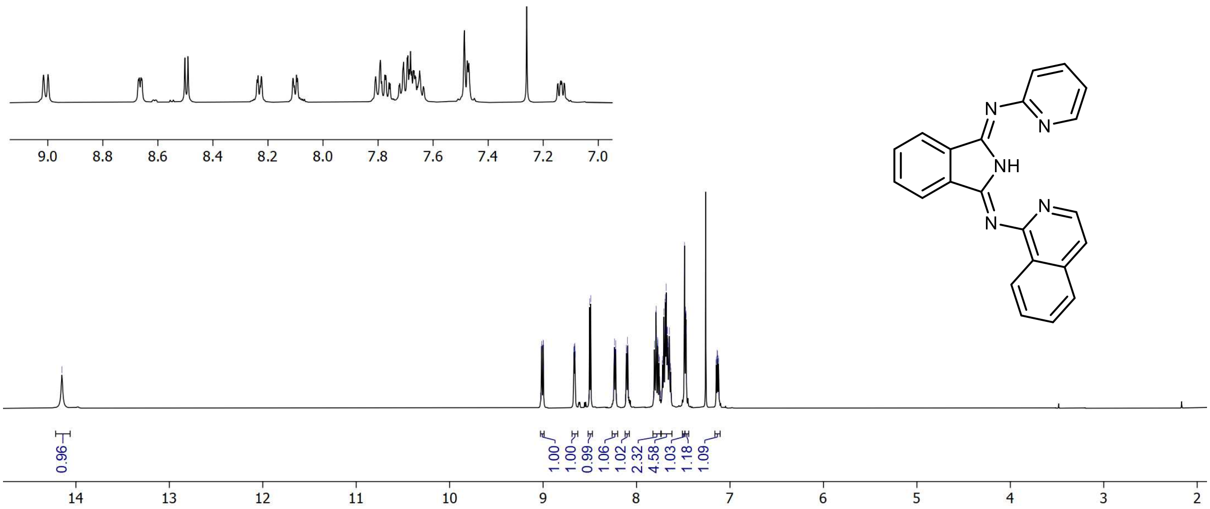

Figure S17. ^{1}H NMR spectrum of **HL^{4}** in $CDCl_3$ (500 MHz). Inset shows aromatic region.

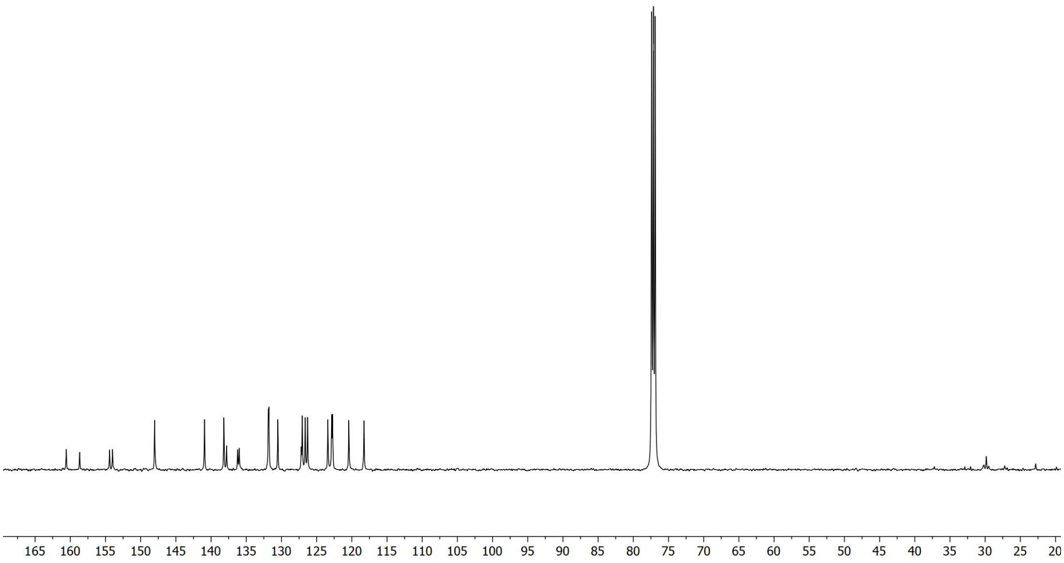

Figure S18. ^{13}C NMR spectrum of **HL^{4}** in $CDCl_3$ (500 MHz).

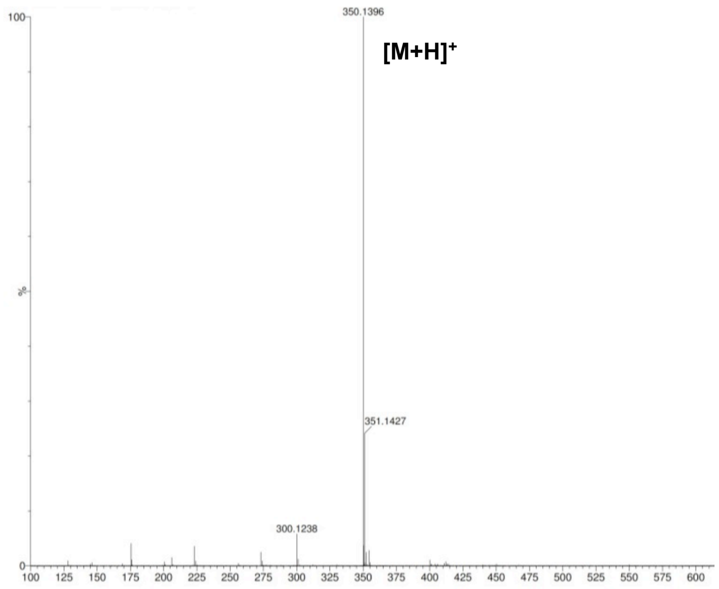

Figure S19. HRMS spectrum of **HL^4**.

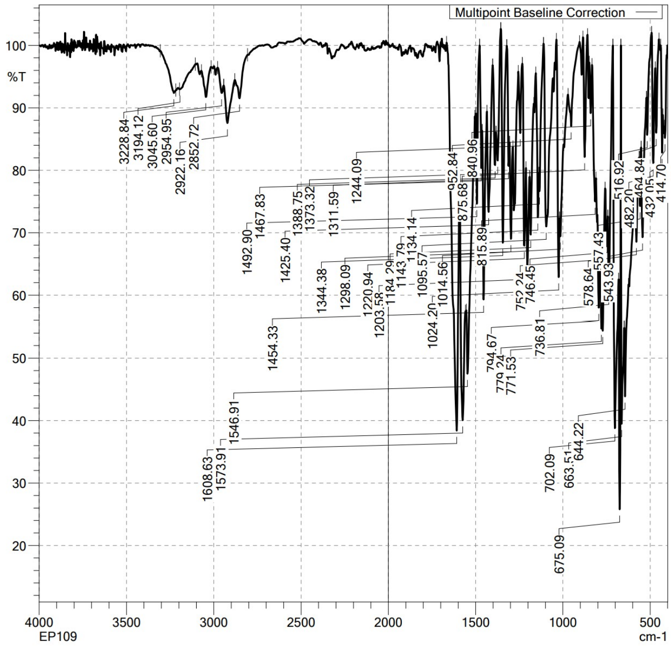

Figure S20. Infrared spectrum of **HL^4**.

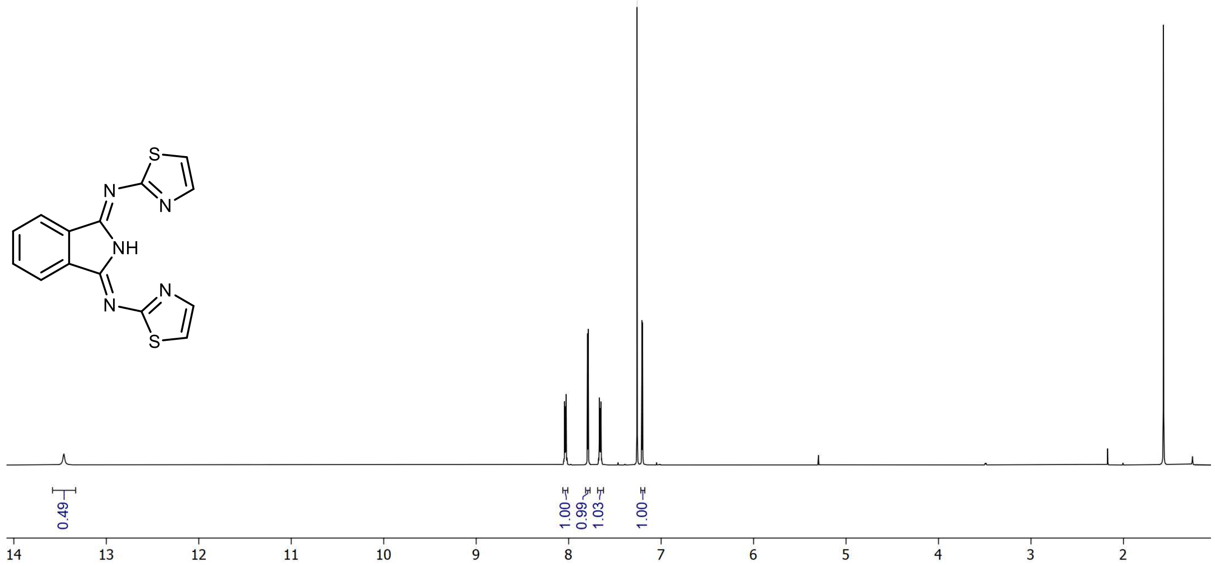

Figure S21. ^{1}H NMR spectrum of **HL^{6}** in $CDCl_3$ (500 MHz).

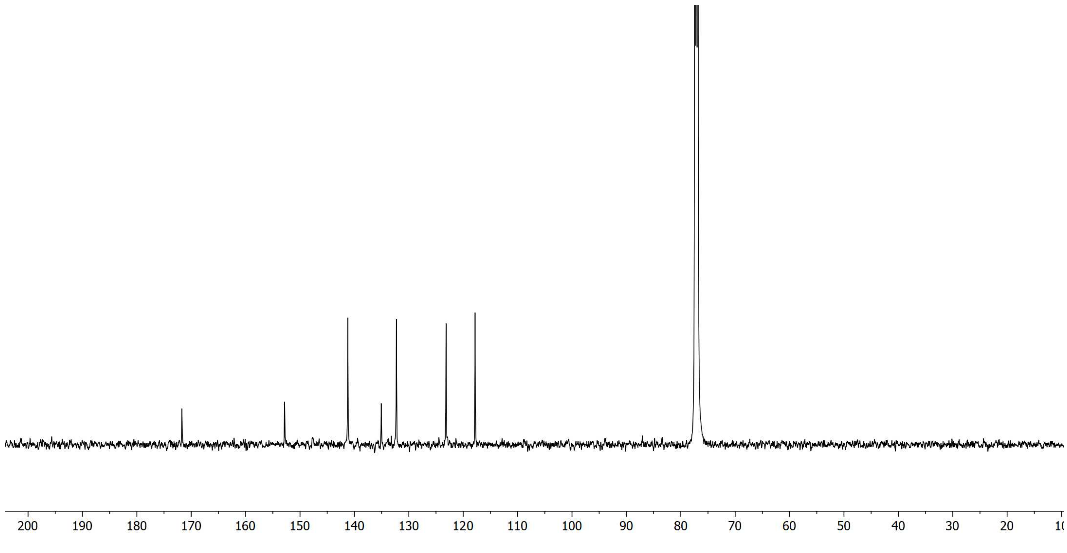

Figure S22. ^{13}C NMR spectrum of **HL^{6}** in $CDCl_3$ (500 MHz).

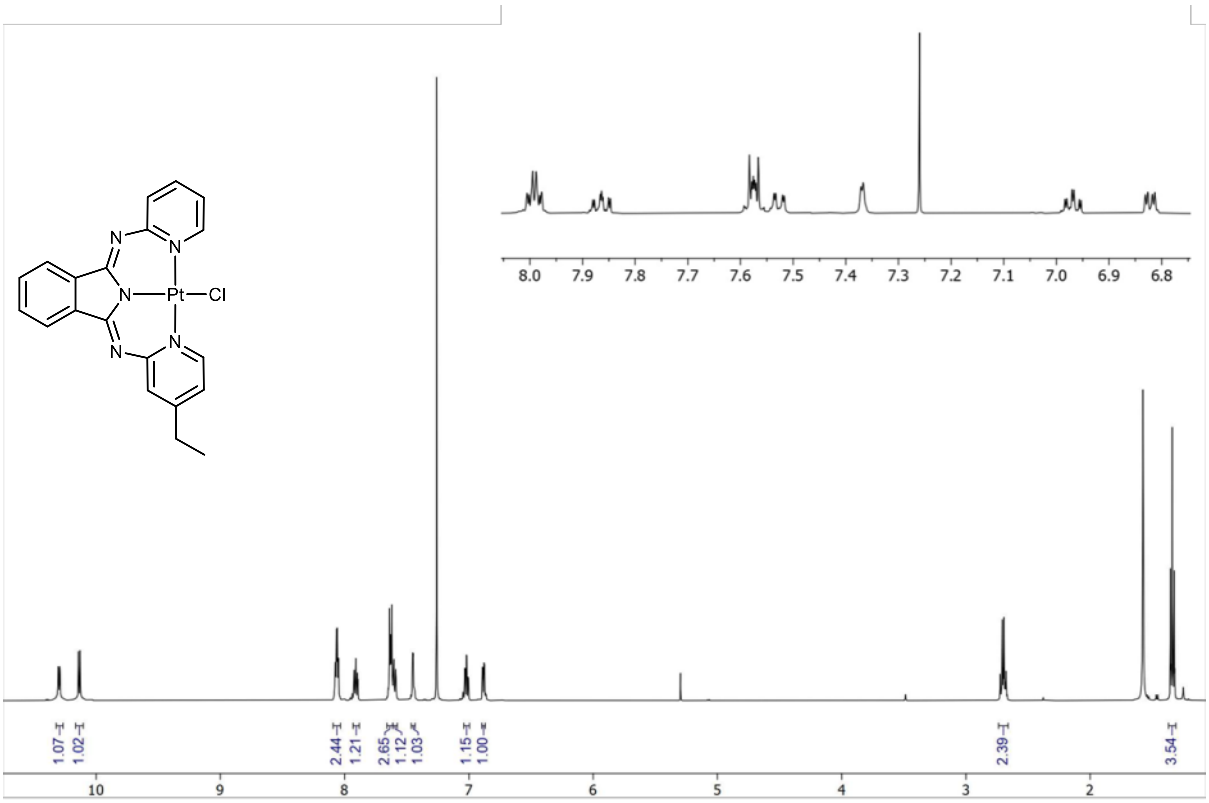

Figure S23. ^{1}H NMR spectrum of Pt($\mathbf{L^1}$)Cl in $CDCl_3$ (500 MHz). Inset shows aromatic region.

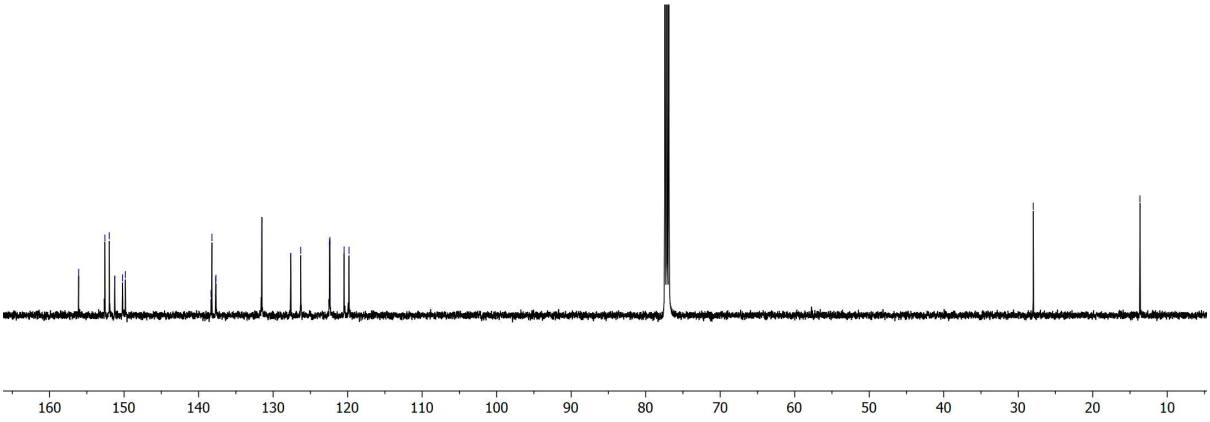

Figure S24. ^{13}C NMR spectrum of Pt($\mathbf{L^1}$)Cl in $CDCl_3$ (500 MHz).

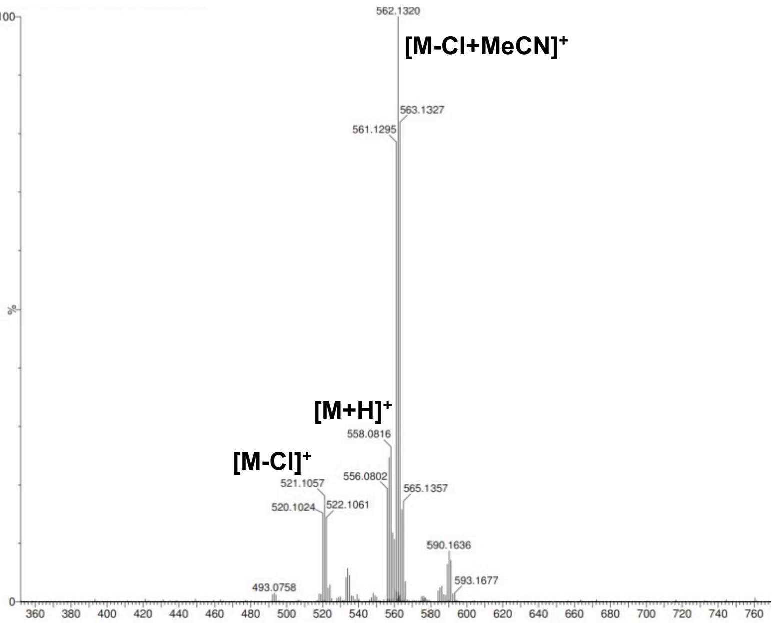

Figure S25. HRMS spectrum of Pt(**L**[1])Cl.

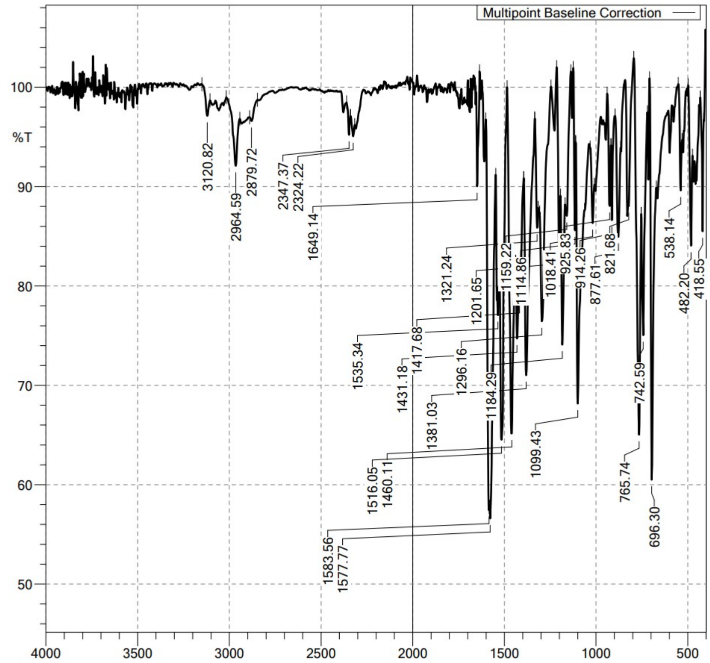

Figure S26. Infrared spectrum of Pt(**L**[1])Cl.

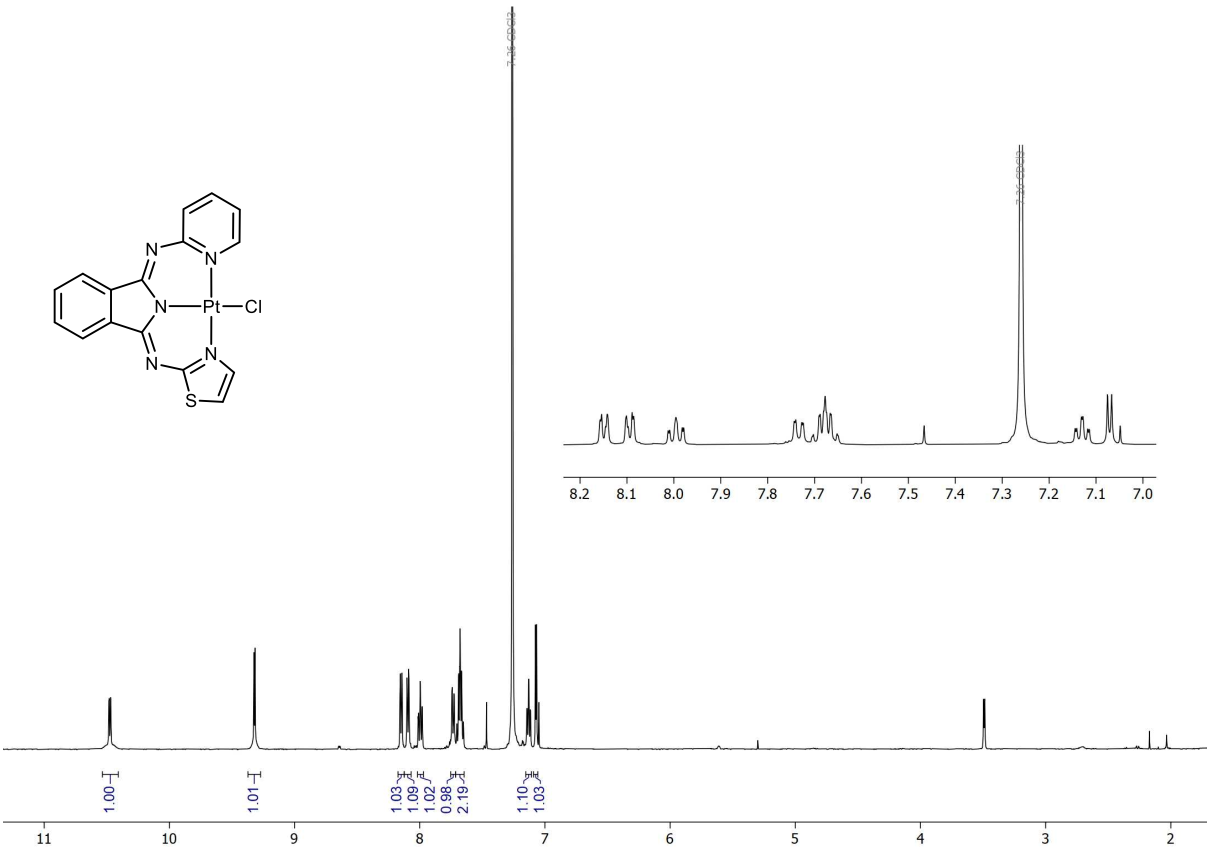

Figure S27. ^{1}H NMR spectrum of Pt(**L**^{2})Cl in $CDCl_3$ (500 MHz). Inset shows aromatic region.

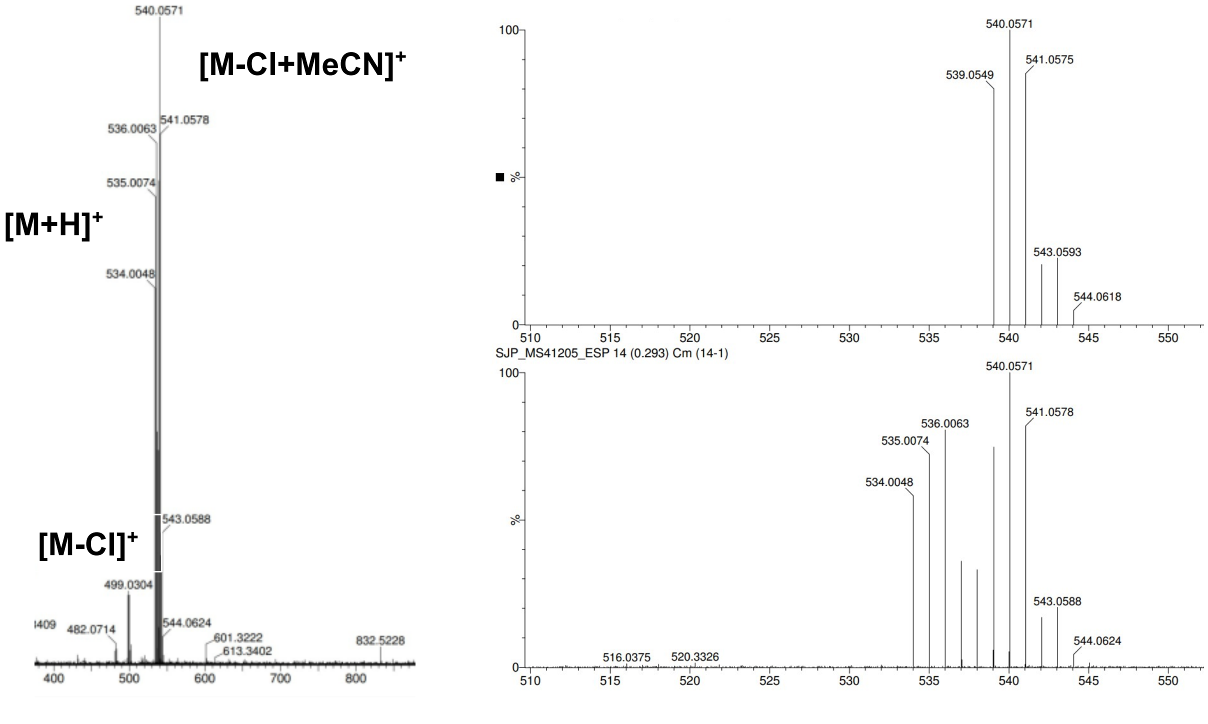

Figure S28. HRMS spectrum of Pt(**L**^{2})Cl. Theoretical (top) Vs Experimental (bottom) High-Resolution Mass spectra showing the overlapping isotope distribution of [Pt(L^{2})MeCN]$^{+}$.

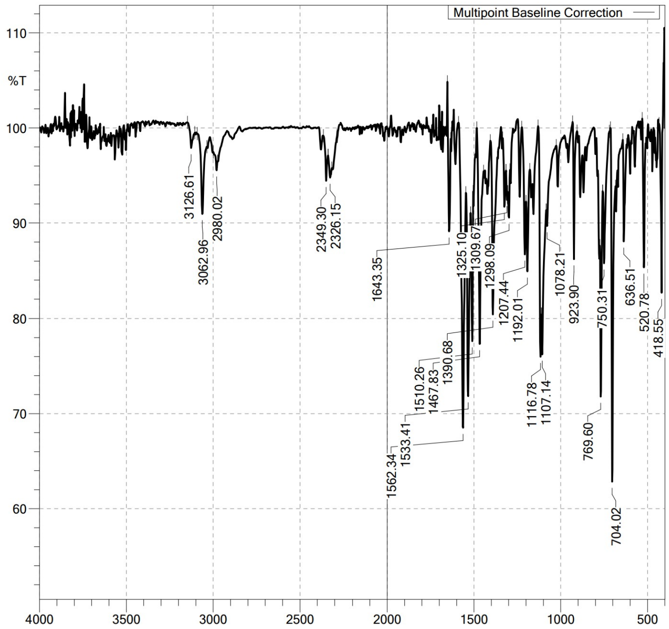

Figure S29. Infrared spectrum of Pt(**L**[2])Cl.

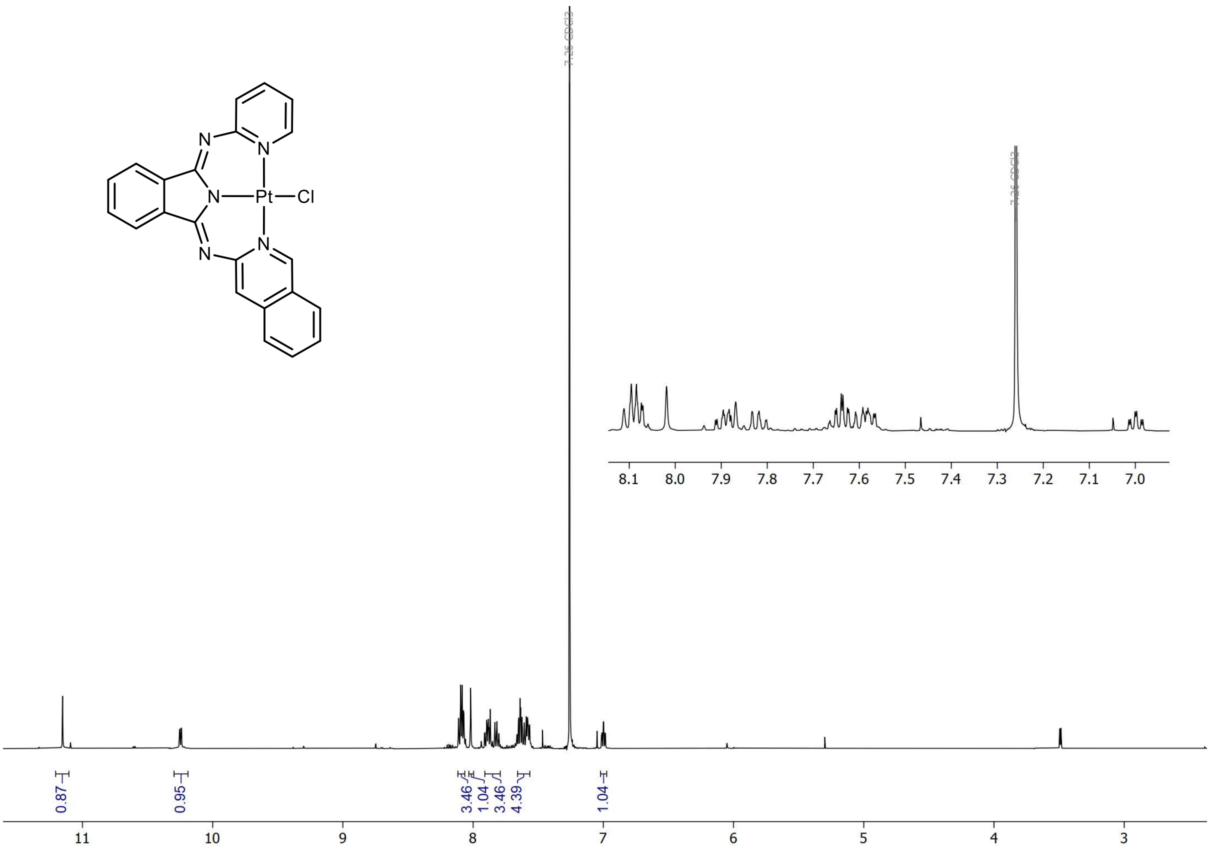

Figure S30. ^{1}H NMR spectrum of Pt(**L**[3])Cl in $CDCl_3$ (500 MHz). Inset shows aromatic region.

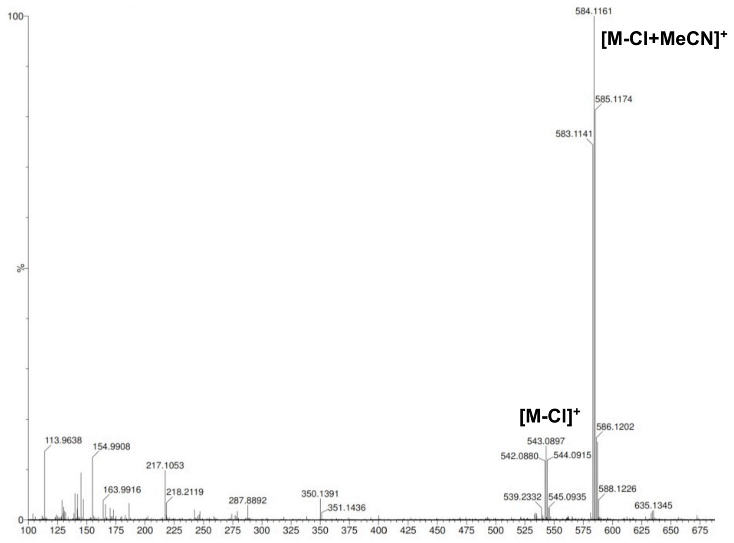

Figure S31. HRMS spectrum of Pt(**L**[3])Cl.

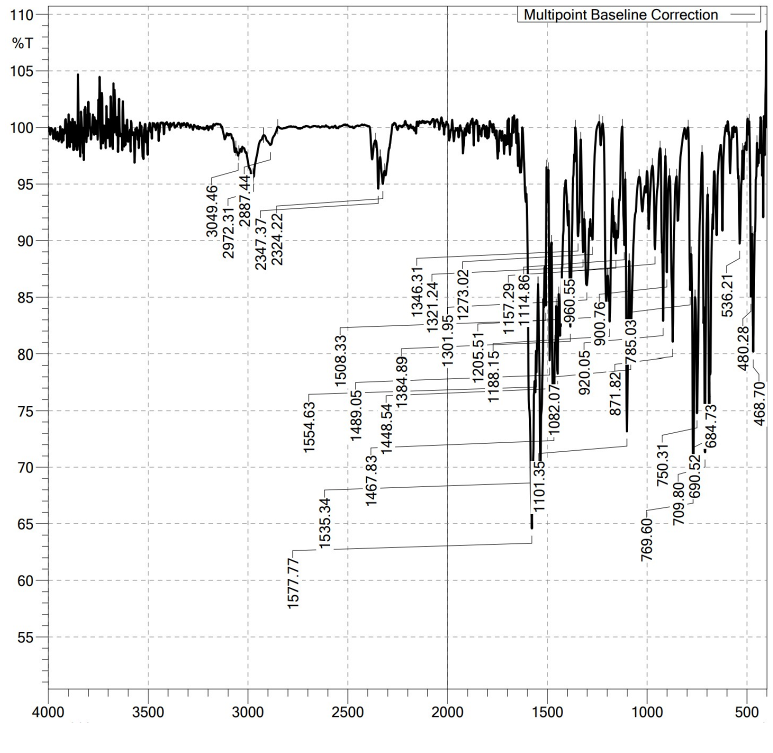

Figure S32. Infrared spectrum of Pt(**L**[3])Cl.

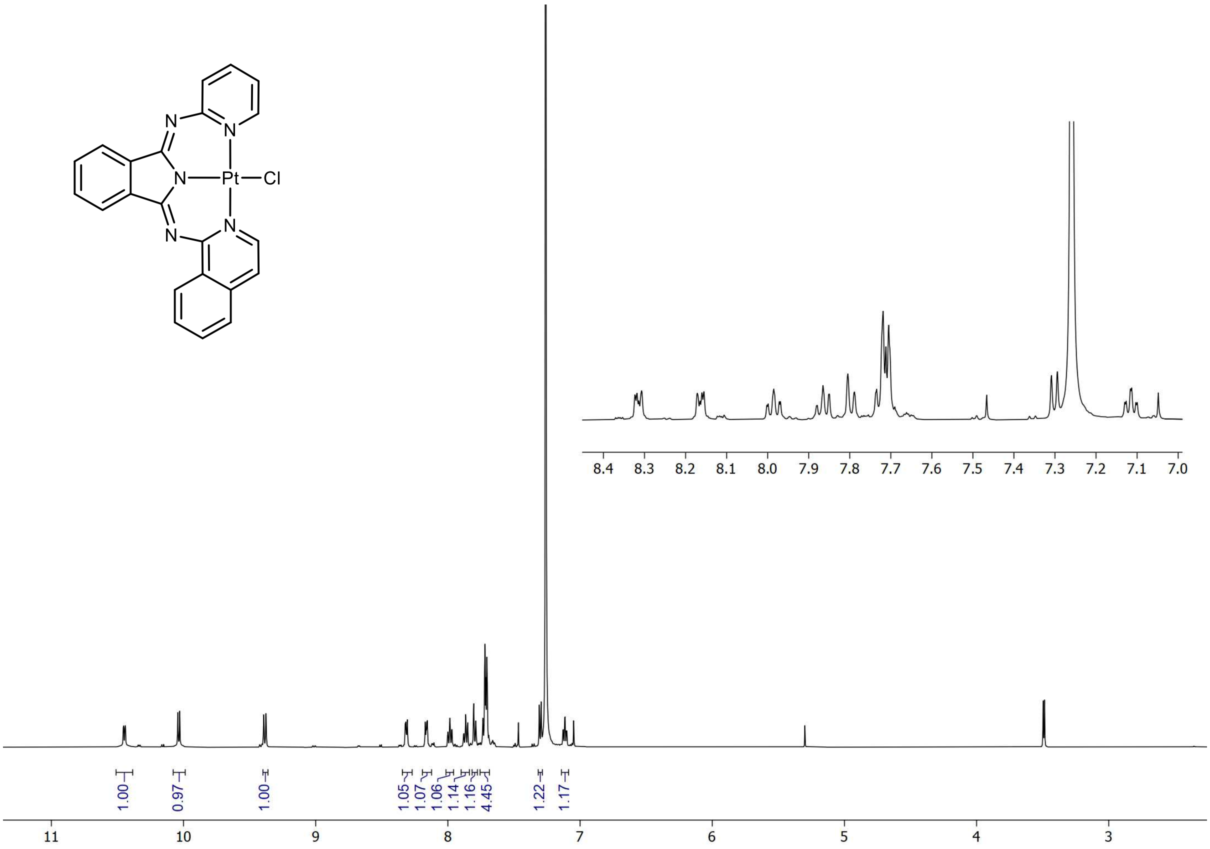

Figure S33. ^{1}H NMR spectrum of Pt(**L⁴**)Cl in $CDCl_3$ (500 MHz). Inset shows aromatic region.

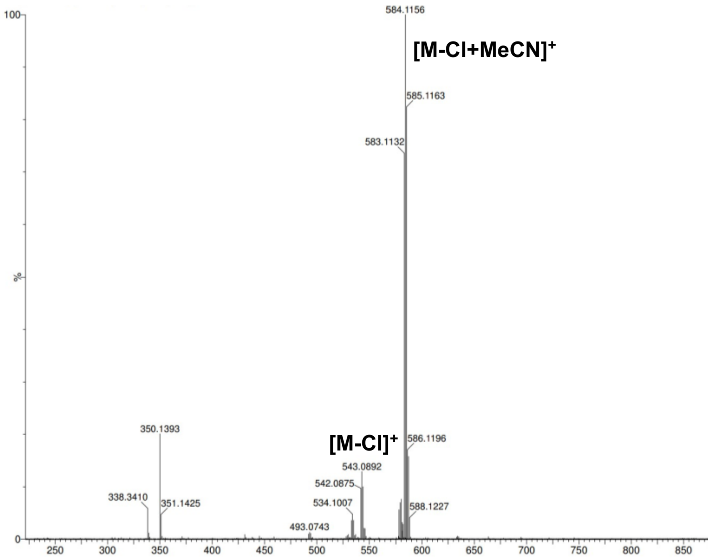

Figure S34. HRMS spectrum of Pt(**L⁴**)Cl.

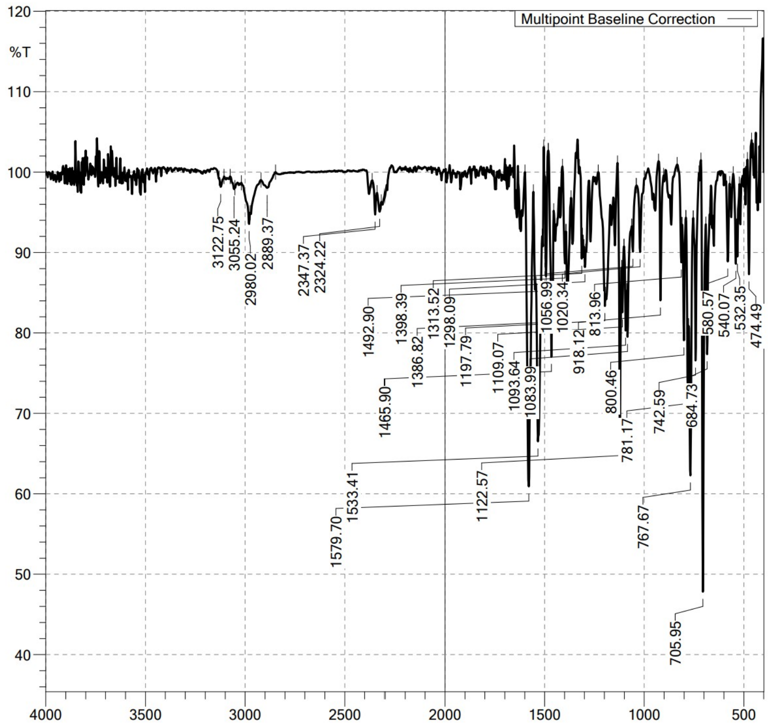

Figure S35. Infrared spectrum of Pt($\mathbf{L^4}$)Cl.

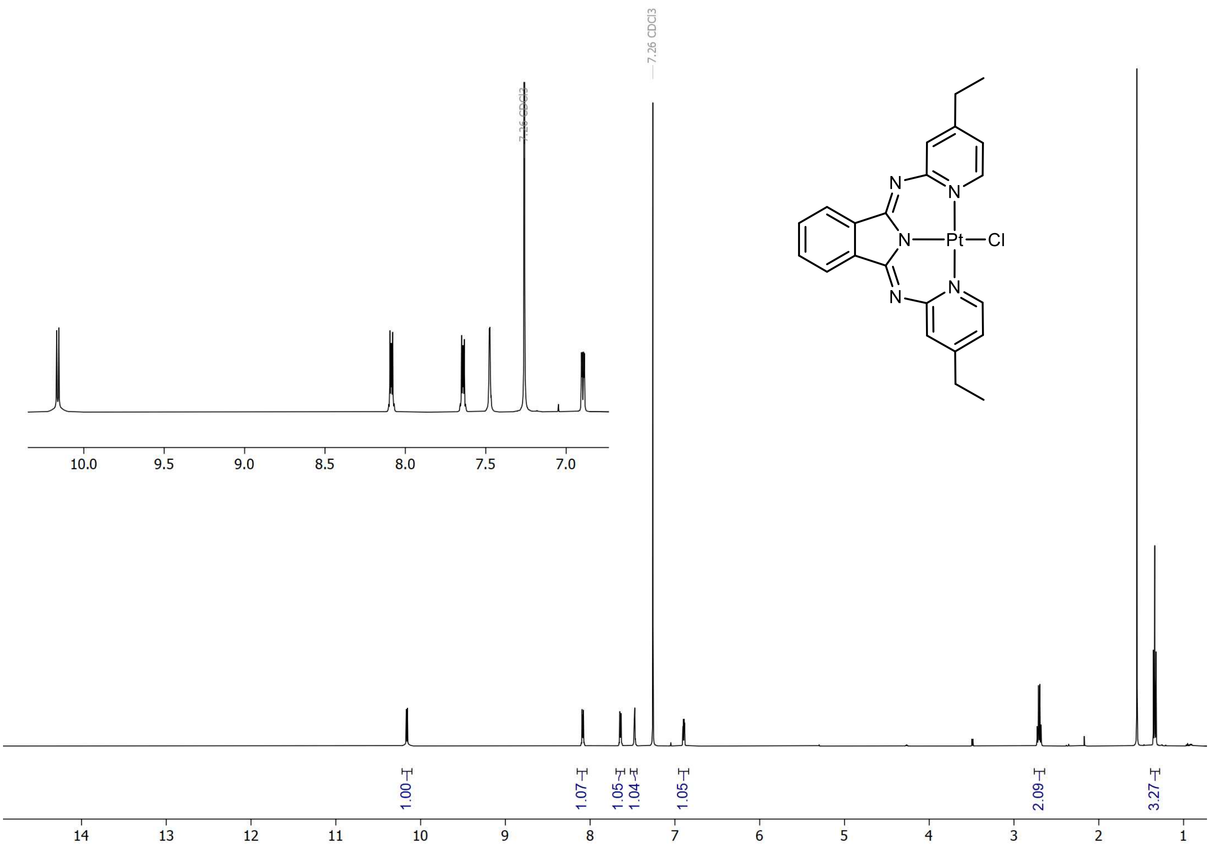

Figure S36. ^{1}H NMR spectrum of Pt($\mathbf{L^5}$)Cl in $CDCl_3$ (500 MHz). Inset shows aromatic region.

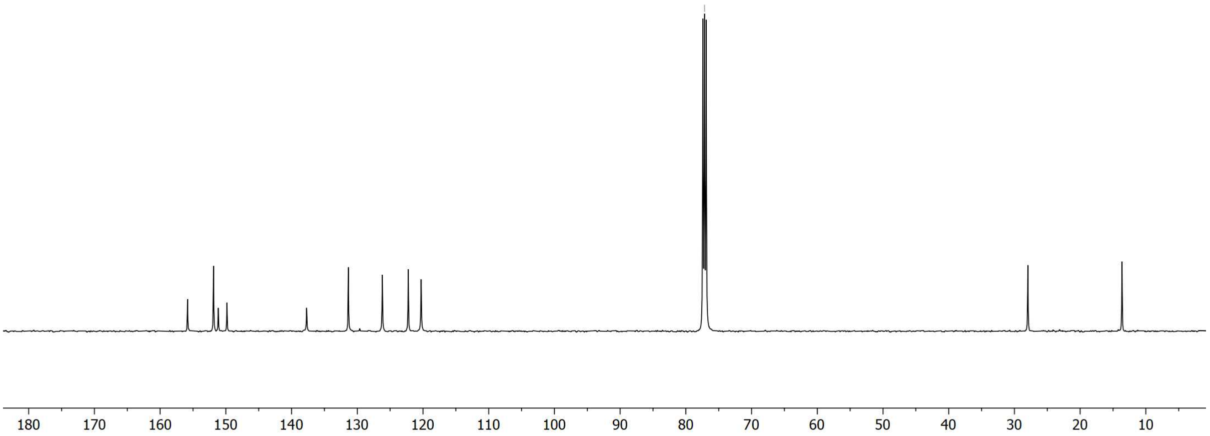

Figure S37. ^{13}C NMR spectrum of Pt(**L**^{5})Cl in $CDCl_3$ (500 MHz).

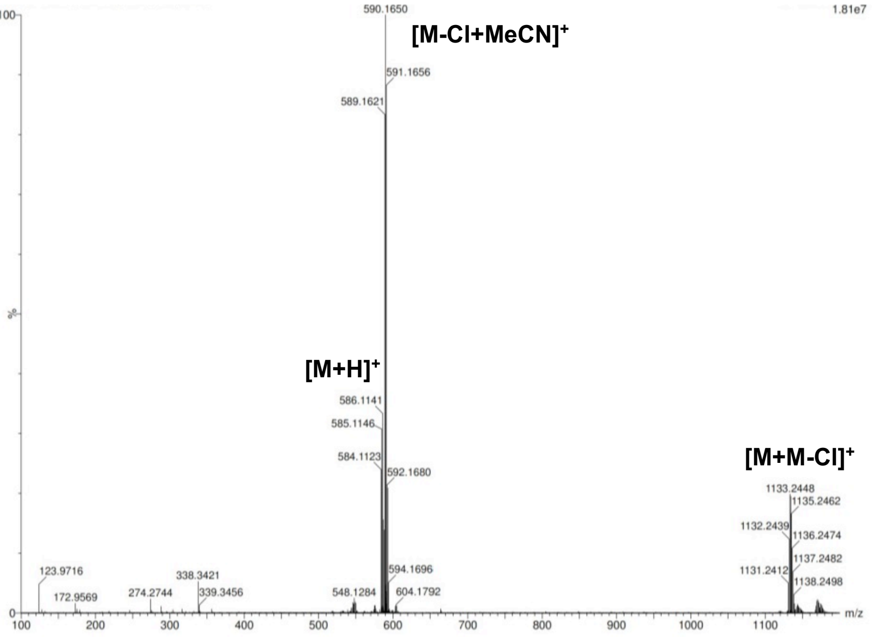

Figure S38. HRMS spectrum of Pt(L^{5})Cl.

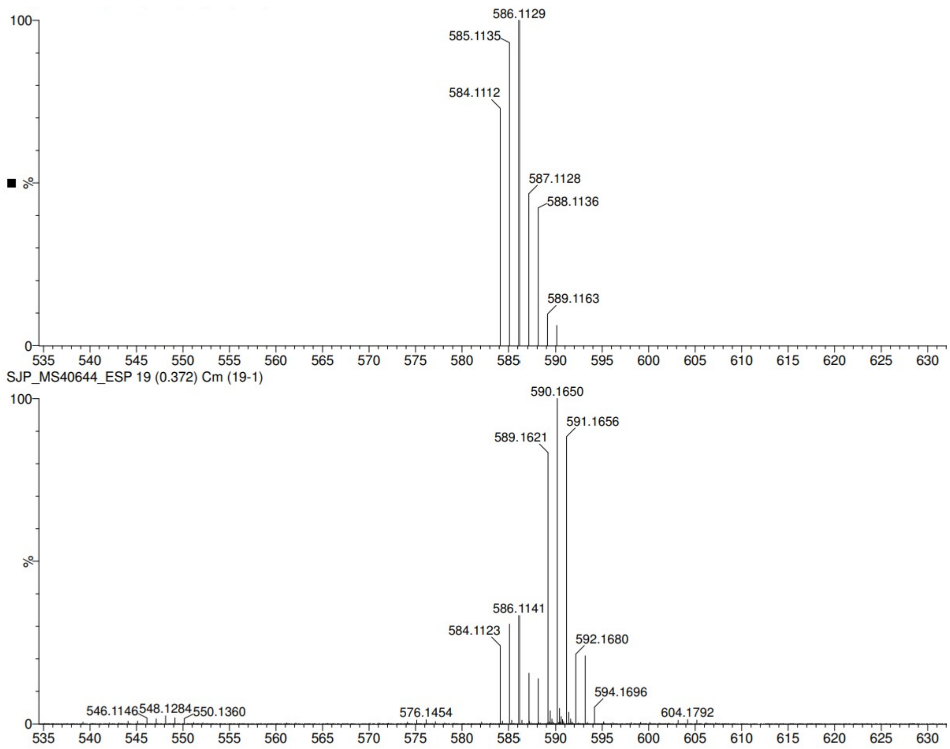

Figure S39. Theoretical (top) Vs Experimental (bottom) High-Resolution Mass spectra showing the overlapping isotope distribution of Pt(**L**[5])Cl ([M+H]$^+$) with [Pt(**L**[5])MeCN]$^+$.

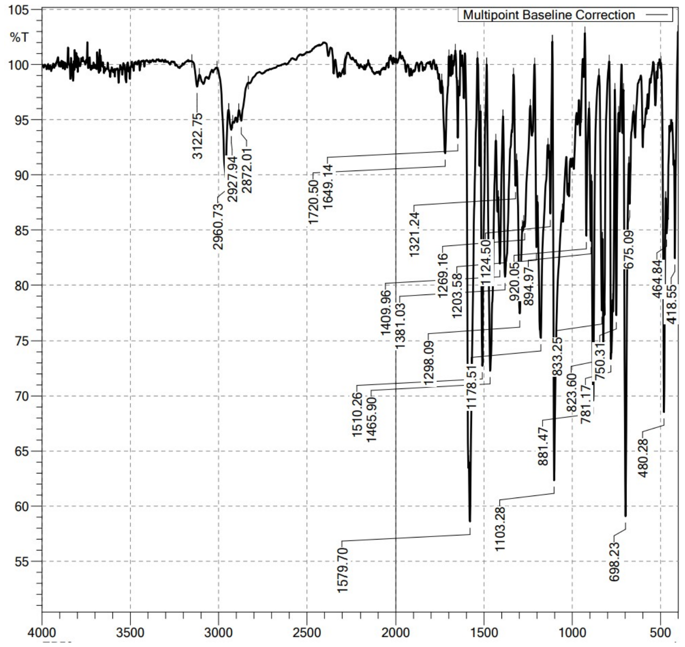

Figure S40. Infrared spectrum of Pt(**L**[5])Cl.

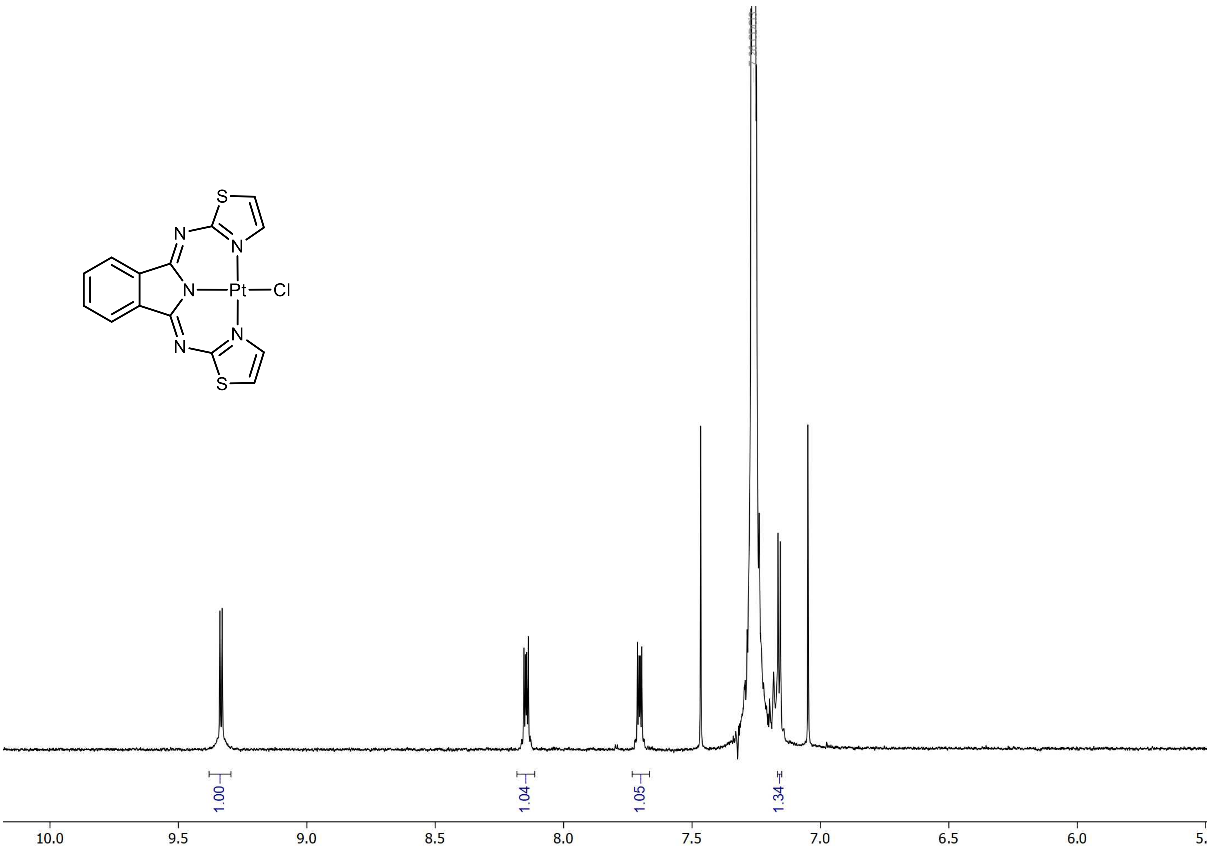

Figure S41. ^{1}H NMR spectrum of Pt(**L**^{6})Cl in $CDCl_3$ (500 MHz).

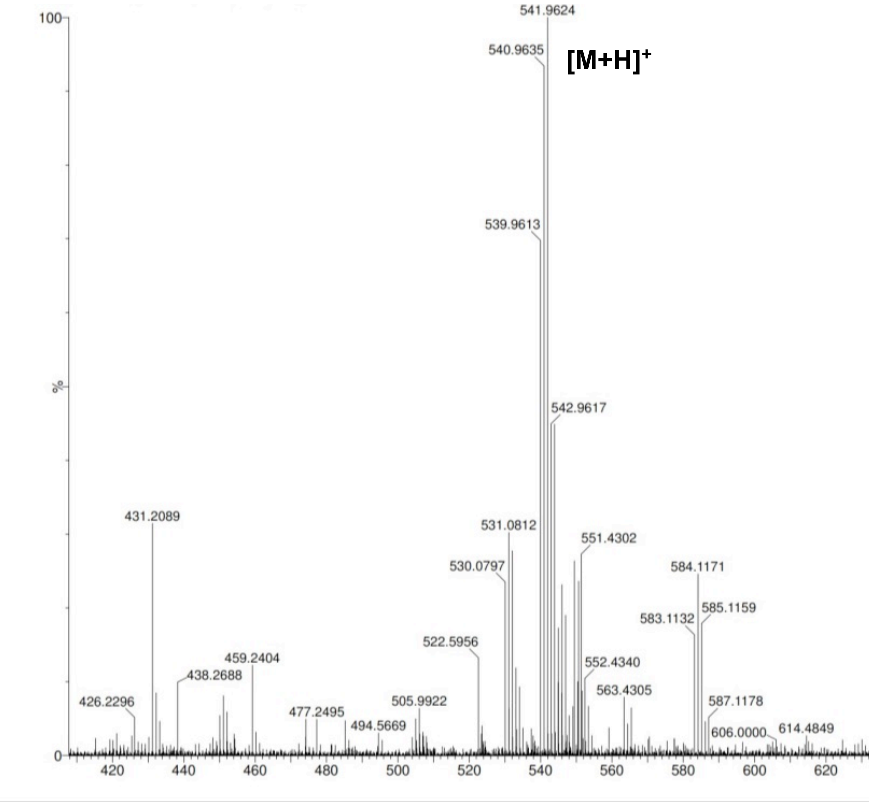

Figure S42. HRMS spectrum of Pt(**L**^{6})Cl.

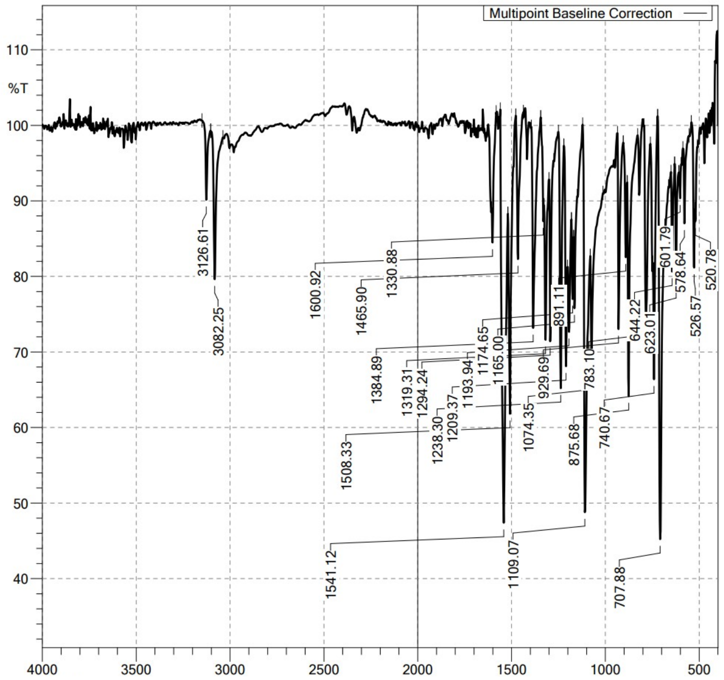

Figure S43. Infrared spectrum of Pt(**L⁶**)Cl.

Table S1. Data collection parameters for the X-ray crystal structure.

	**PtCl(L^5)**
Formula	$C_{22}H_{20}ClN_5Pt$
$D_{calc.}$/ g cm^{-3}	2.007
μ/mm^{-1}	7.405
Formula Weight	584.97
Colour	orange
Shape	rod-shaped
Size/mm^3	0.220×0.060×0.050
T/K	100(2)
Crystal System	triclinic
Space Group	P-1
a/Å	10.8844(2)
b/Å	11.3037(2)
c/Å	17.8105(2)
α/°	103.6830(10)
β/°	94.2190(10)
γ/°	112.3630(10)
V/Å^3	1936.08(6)
Z	4
Z'	2
Wavelength/Å	0.71075
Radiation type	Mo K_α
Θ_{min}/°	2.034
Θ_{max}/°	33.804
Measured Refl's.	63365
Indep't Refl's	13519
Refl's I≥2 σ(I)	11613
R_{int}	0.0337
Parameters	527
Restraints	0
Largest Peak	1.296
Deepest Hole	-1.479
GooF	1.058
wR_2 (all data)	0.0457
wR_2	0.0442
R_1 (all data)	0.0292
R_1	0.0209

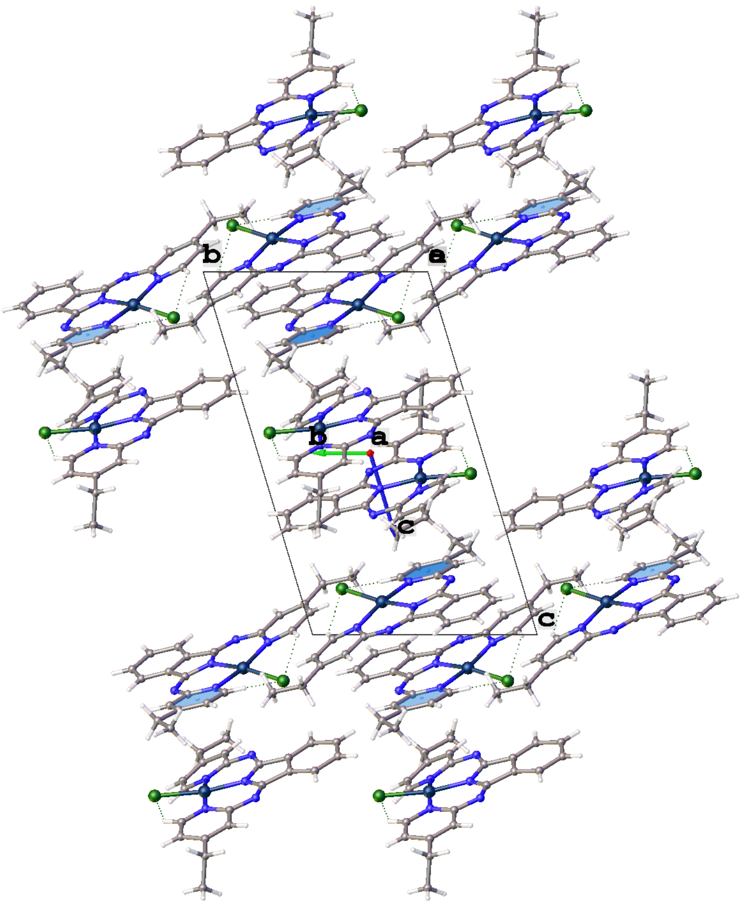

Figure S44. Packing diagram obtained from the X-ray structure of Pt($\mathbf{L}^5$)Cl.

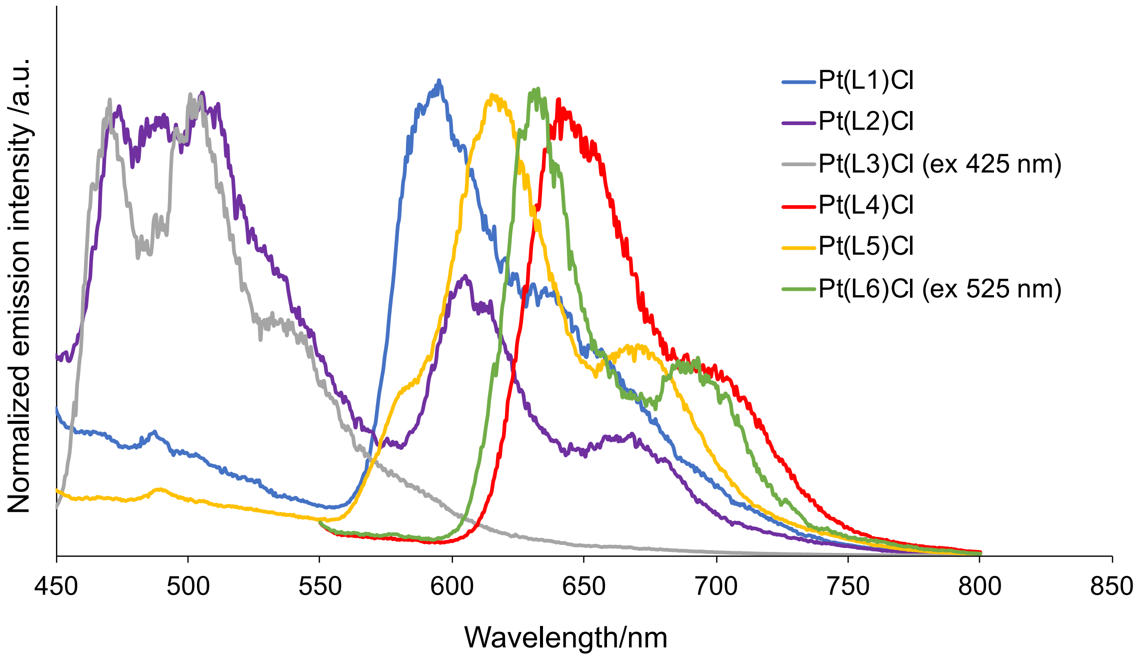

Figure S45. Total emission spectra recorded at 77 K of Pt($\mathbf{L}^n$)Cl (4:1, chloroform/toluene).
